# The oncometabolite 2-hydroxyglutarate activates the mTOR signalling pathway

**DOI:** 10.1038/ncomms12700

**Published:** 2016-09-14

**Authors:** Mélissa Carbonneau, Laurence M. Gagné, Marie-Eve Lalonde, Marie-Anne Germain, Alena Motorina, Marie-Christine Guiot, Blandine Secco, Emma E. Vincent, Anthony Tumber, Laura Hulea, Jonathan Bergeman, Udo Oppermann, Russell G. Jones, Mathieu Laplante, Ivan Topisirovic, Kevin Petrecca, Marc-Étienne Huot, Frédérick A. Mallette

**Affiliations:** 1 Département de Biochimie et Médecine Moléculaire, Université de Montréal, C.P. 6128, Succursale Centre-Ville, Montréal, Quebec, Canada H3C 3J7; 2 Chromatin Structure and Cellular Senescence Research Unit, Maisonneuve-Rosemont Hospital Research Centre, Montréal, Quebec, Canada H1T 2M4; 3 Centre de Recherche sur le Cancer de l'Université Laval; Départements de Biologie moléculaire, biochimie médicale et pathologie, Université Laval, Québec City, Quebec, Canada G1V 0A6; 4 Brain Tumour Research Centre, Montreal Neurological Institute and Hospital, Department of Neurology and Neurosurgery, McGill University, 3801 University Street, Montreal, Quebec, Canada H3A 2B4; 5 Division of Neuropathology, Department of Pathology, McGill University, Montreal, Quebec, Canada H3A 2B4; 6 Institut universitaire de cardiologie et de pneumologie de Québec, 2725 chemin Sainte-Foy, Québec City, Quebec, Canada G1V 4G5; 7 Département de Médecine, Faculté de Médecine, Université Laval, Québec City, Quebec, Canada G1V 0A6; 8 Goodman Cancer Research Centre, McGill University, Montréal, Quebec, Canada H3G 1Y6; 9 Department of Physiology, McGill University, Montréal, Quebec, Canada H3G 1Y6; 10 Structural Genomics Consortium, University of Oxford, Old Road Campus, Roosevelt Drive, Headington OX3 7DQ, UK; 11 Target Discovery Institute, University of Oxford, NDM Research Building, Roosevelt Drive, Oxford OX3 7LD, UK; 12 Lady Davis Institute for Medical Research, Jewish General Hospital, 3755 Côte-Ste-Catherine Road, Montréal, Quebec, Canada H3T 1E2; 13 Department of Oncology, McGill University, 546 Pine Avenue West, Montréal, Quebec, Canada H2W 1S6; 14 Botnar Research Center, NIHR Oxford Biomedical Research Unit, Nuffield Department of Orthopaedics, Rheumatology & Musculoskeletal Sciences, Oxford OX3 7LD, UK; 15 CHU de Québec–Axe Oncologie (Hôtel-Dieu de Québec), Québec City, Quebec Canada G1R 3S3; 16 Département de Médecine, Université de Montréal, C.P. 6128, Succursale Centre-Ville, Montréal, Quebec, Canada H3C 3J7

## Abstract

The identification of cancer-associated mutations in the tricarboxylic acid (TCA) cycle enzymes isocitrate dehydrogenases 1 and 2 (IDH1/2) highlights the prevailing notion that aberrant metabolic function can contribute to carcinogenesis. IDH1/2 normally catalyse the oxidative decarboxylation of isocitrate into α-ketoglutarate (αKG). In gliomas and acute myeloid leukaemias, IDH1/2 mutations confer gain-of-function leading to production of the oncometabolite *R*-2-hydroxyglutarate (2HG) from αKG. Here we show that generation of 2HG by mutated IDH1/2 leads to the activation of mTOR by inhibiting KDM4A, an αKG-dependent enzyme of the Jumonji family of lysine demethylases. Furthermore, KDM4A associates with the DEP domain-containing mTOR-interacting protein (DEPTOR), a negative regulator of mTORC1/2. Depletion of KDM4A decreases DEPTOR protein stability. Our results provide an additional molecular mechanism for the oncogenic activity of mutant IDH1/2 by revealing an unprecedented link between TCA cycle defects and positive modulation of mTOR function downstream of the canonical PI3K/AKT/TSC1-2 pathway.

## Abstract (web summary)

Oncogenic mutations of isocitrate dehydrogenases 1 and 2 result in the production of the oncometabolite R-2-hydroxyglutarate. Here the authors show that the oncometabolite promotes mTOR activation in a PTEN/PI3K-independent manner by regulating DEPTOR stability via inhibition of KDM4A activity.

## Graphical abstract

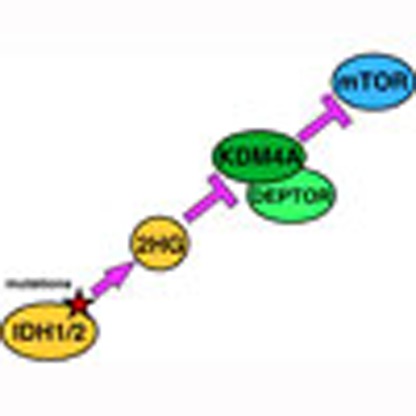

It is now evident that aberration of normal metabolic function represents a hallmark of tumorigenesis^1^. For example, mutation, deletion or silencing of genes encoding tricarboxylic acid cycle (TCA cycle) proteins, including isocitrate dehydrogenases 1 and 2 (IDH1/2), are frequent in acute myeloid leukaemias and brain cancers (WHO grade 2 and 3 astrocytomas, oligodendrogliomas and secondary glioblastomas)^2^,^3^. IDH1/2 mutations occur predominantly at arginine 132 for IDH1, and arginine 140 or 172 for IDH2 (refs 2, 3), in each case resulting in a gain of function that promotes conversion of αKG to 2HG (refs 4, 5). These latter metabolites share high structural similarity, allowing 2HG to bind and inhibit αKG-dependent enzyme activity including that of the Jumonji lysine demethylases family and the Ten-eleven translocation (TET) family of 5-methylcytosine hydroxylases^6^,^7^.

Jumonji family members require αKG as co-substrate to catalyse the removal of methyl moieties on lysine. Conversely, 2HG has been shown to effectively inhibit this family of enzymes^6^,^7^. Jumonji proteins are oxygenases catalysing demethylation of mono-, di- or tri-methylated lysines^8^,^9^. To date, more than 30 Jumonji proteins have been identified, which carry different secondary domains in addition to their common JmjC catalytic domain. These enzymes are classified into numerous sub-families according to sequence homology and substrate specificity. The KDM4 sub-family comprises four members (KDM4A-D) which are Fe(II)- and α-ketoglutarate-dependent enzymes possessing a JmjC catalytic domain^8^,^9^. KDM4A-C display similar structures including JmjN-, tandem PHD-, and tandem Tudor-domains and each catalyses demethylation of di- and tri-methylated H3K9 and H3K36. On the other hand, KDM4D lacks the PHD and tandem Tudor domains and is specific for H3K9(me2/3) (refs 10, 11). KDM4A is involved in the regulation of many cellular processes including cell cycle progression, DNA damage signalling and Ras-induced senescence^12^,^13^,^14^.

The mammalian (mechanistic) Target Of Rapamycin (mTOR) is a serine/threonine kinase belonging to the phosphoinositide 3-kinase (PI3K)-related kinase (PIKK) family. mTOR associates with either Raptor or Rictor to form distinct complexes, that is, mTORC1 and mTORC2, respectively, both of which are regulated by growth factors, amino acids and energy levels^15^,^16^,^17^,^18^. PRAS40 is recovered within the mTORC1 complex, while mSin1 and Protor1/2 are present in the mTORC2 complex^19^,^20^,^21^,^22^. mLST8 and DEPTOR, an inhibitor of mTOR, are common to both mTORC1 and mTORC2 (refs 23, 24). Among the cellular processes regulated by mTORC1 are cell growth and autophagy, while mTORC2 regulates cell survival and cytoskeleton organisation (for a review, see ref. 25). mTORC1/2 are frequently activated in human cancers via mutation of upstream negative regulators such as *PTEN* or *TSC2.* Following mTOR activation, DEPTOR is phosphorylated by mTOR, which in turn leads to phosphorylation of DEPTOR by CK1 (refs 26, 27). These phosphorylation events are recognized by SCF^β-TrCP^ ubiquitin ligase, resulting in DEPTOR polyubiquitination and proteolytic degradation^26^,^27^,^28^. Following its activation by mTORC1, p70S6K can also phosphorylate DEPTOR, thus facilitating β-TrCP binding^28^.

Herein we provide intriguing evidence for a novel oncogenic mechanism regulated by mutations of IDH1/2, whereby 2HG-induced KDM4A inhibition promotes mTOR activation. We show that KDM4A specifically interacts with DEPTOR, a negative regulator of both mTORC1 and mTORC2. KDM4A depletion/inactivation results in drastic reduction of DEPTOR levels, while KDM4A overexpression reduces DEPTOR ubiquitination by β-TrCP1. We also show that 2HG-mediated inhibition of KDM4A directly affects the stability of DEPTOR, leading to mTOR activation independently of the PI3K/AKT/TSC1-2 pathway. This newly revealed PTEN-independent mode of mTORC1/2 activation via the mutated IDH/2HG/KDM4A/DEPTOR pathway may provide an additional molecular mechanism to explain the oncogenic activity of IDH1/2 mutations in glioma and other cancers.

## Results

### *IDH1/2* and *PTEN* mutations are mutually exclusive

**Figure 1:**
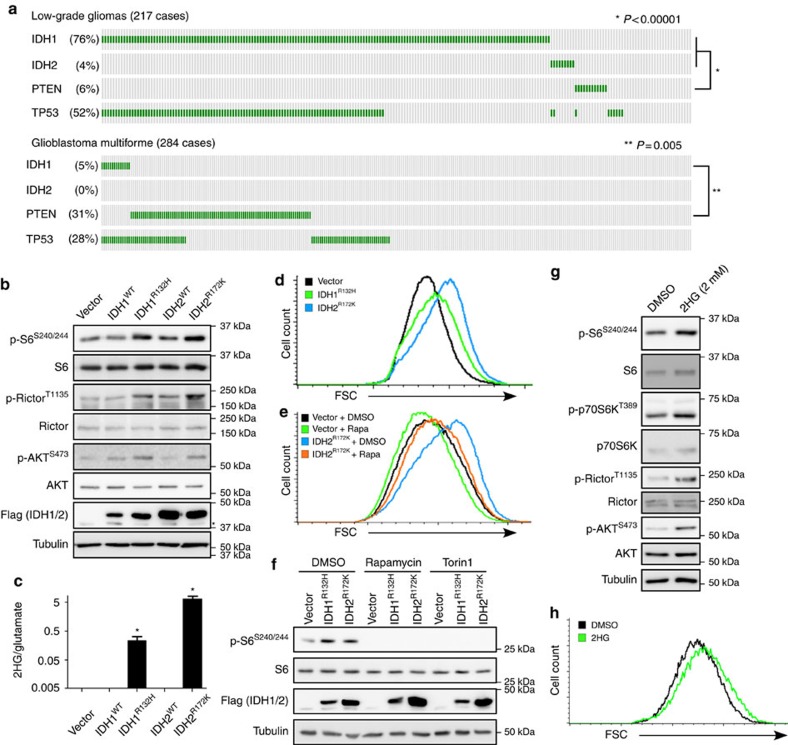
Activation of mTOR signalling by 2HG-producing IDH1/2 gain-of-function mutations. (**a**) *IDH1/2* and *PTEN* mutations are mutually exclusive in low-grade gliomas and glioblastomas. Data were obtained from The Cancer Genome Atlas (TCGA). Each vertical lane represents one patient; green lines depict the presence of a mutation. Statistical analysis was performed using the Fisher's exact test. (**b**) mTOR pathway activation in MEFs p53^−/−^ cells expressing wt versus mutant IDH. Flag-tagged IDH was retrovirally transduced into MEFs p53^−/−^ cells, and whole-cell lysates were harvested at 9 days post selection. Asterisk denotes a nonspecific band recognized by the antibody. (**c**) Intracellular accumulation of 2HG on IDH1/2 mutant expression as in **b**. 2HG levels were normalized against glutamate levels. Asterisks denote a statistical difference between IDH1/2 mutated cells and empty vector cells, two-sided *t*-test *P*<0.0001 (graph represents three independent experiments). Error bars represent standard deviation. (**d**) Increased cell size associated with mutated *IDH1/2* expression. FSC measurement in FACS analysis demonstrates the shift in cell size in MEFs p53^−/−^ cells expressing IDH1^R123H^ (green line) or IDH2^R172K^ (blue line) compared with empty vector-infected cells (black line). A total 250,000 cells were counted for each condition and graph is representative of four independent experiments. (**e**) Inhibition of mTORC1 rescues the increased cell size in IDH2^R172K^ mutant cells. MEFs p53^−/−^ cells expressing IDH2^R172K^ or empty vector were treated with 25 nM rapamycin for 16 h before FACS analysis. A total 250,000 cells were counted for each condition. (**f**) Rapamycin or Torin1 treatment prevents mTORC1 activation in cells expressing IDH1/2 mutants. MEFs p53^−/−^ cells expressing IDH1/2 mutants were treated with 100 nM rapamycin or 250 nM Torin1 for 24 h and whole-cell lysates blotted for markers of mTORC1 activation. (**g**) 2HG stimulates the mTOR pathway. MEFs p53^−/−^ were treated with 2 mM of octyl-2HG for 4 h, and whole-cell extracts analysed by western blot. (**h**) Octyl-2HG treatment increases cell size. MEFs p53^−/−^ cells were treated with 1 mM octyl-2HG for 8 h before FCS analysis by FACS. A total 25,000 cells were counted for each condition and graph is representative of two independent experiments.

Towards elucidating the oncogenic properties of mutated *IDH1/2*, we analysed *The Cancer Genome Atlas* (TCGA) and deduced that mutations in *IDH1/2* and *PTEN* (Phosphatase and TENsin homologue), an important tumour suppressor frequently disrupted in many cancers, display mutual exclusivity in low-grade glioma (*P*<0.00001; Fisher's exact test) and glioblastoma multiforme (*P*=0.005; Fisher's exact test; Fig. 1a). On the other hand, as previously reported^29^,^30^, mutations in the tumour suppressor *tp53* often co-occur with *IDH1/2* mutations in low-grade glioma (*P*<0.0001; Fisher's exact test) and glioblastoma multiforme (*P*<0.00001; Fisher's exact test) (Fig. 1a). Thus it appears that major cancer networks can be strongly influenced by *IDH1/2* gene status. The mutual exclusivity between *IDH1/2* and *PTEN* mutations suggests that abrogation of *PTEN* function might not confer a growth advantage to tumours carrying *IDH1/2* mutations, allowing speculation that *IDH1/2* mutations mimic *PTEN* loss or inactivation. PTEN governs multiple cellular functions such as survival, growth and energy metabolism by suppressing the phosphoinositide 3-kinase (PI3K)/AKT/mTOR pathway. Oncogenic activation of mTOR stimulates critical processes required for cancer cell survival, proliferation, angiogenesis and metastasis^25^. Importantly, even though no *PTEN* mutations were observed in *IDH1/2*-mutated gliomas, the mTOR pathway is nonetheless frequently activated in these tumours^31^.

### IDH1/2 mutations and 2HG activate mTOR signalling

The above considerations prompted us to postulate the existence of a PTEN-independent mode of mTOR pathway activation by oncogenic *IDH1/2*, which might constitute an important determinant in glioma development. We first evaluated the possibility that the mTOR pathway is actually regulated by oncogenic *IDH1/2*. Murine embryonic fibroblasts (MEFs) retrovirally transduced with cancer-associated IDH1^R132H^ or IDH2^R172K^, but not with wild-type counterparts, exhibited increased mTORC1 and mTORC2 signalling as highlighted by enhanced phosphorylation of p70S6K, S6 and Rictor, and AKT, respectively (Fig. 1b and Supplementary Fig. 1a). The extent of mTORC1 stimulation correlated with intracellular levels of the oncometabolite *R*-2-hydroxyglutarate (2HG) produced by mutant IDH1 and IDH2 (Fig. 1c). Intracellular accumulation of 2HG in mutated IDH1/2-expressing cells is not associated with decreased levels of αKG levels (Supplementary Fig. 1b), which has been shown, in different studies, to either inhibit^32^ or stimulate^33^,^34^,^35^ mTOR activity.

It has been shown that mTOR activity is a major determinant of cell size^36^. Therefore, to probe the physiological response to mutant IDH1/2-mediated activation of mTOR signalling, we compared the effects of IDH1^WT^, IDH1^R132H^, IDH2^WT^ and IDH2^R172K^ expression in MEFs on cell size using flow cytometry. Cells expressing mutated IDH1/2 were larger than control counterparts (Fig. 1d and Supplementary Fig. 1c,d), and this increase correlated with intracellular levels of 2HG (Fig. 1c). Moreover, treatment of IDH2^R172K^-expressing cells with the mTORC1 inhibitor rapamycin mitigated the effect on cell size (Fig. 1e). In addition, treatment with rapamycin or the mTORC1/2 inhibitor Torin1 abrogated S6 phosphorylation on serines 240 and 244 in IDH1/2 mutant-expressing cells, confirming the activation of mTORC1-dependent p70S6K by 2HG-producing mutants (Fig. 1f). Expression of IDH1^R132H^ in cells treated with AGI-5198, an inhibitor of mutated IDH1 (ref. 37), failed to activate mTORC1 (Supplementary Fig. 1e), suggesting that oncogenic IDH1-mediated production of 2HG is responsible for mTOR stimulation.

To directly evaluate whether 2HG is responsible for activation of mTORC1/2, we synthesized cell permeable 1-octyl-D-2-hydroxyglutarate (octyl-2HG) (Supplementary Fig. 1f)^38^. Treatment of cells with octyl-2HG led to activation of mTORC1/2, as illustrated by increased phosphorylation of p70S6K, S6, Rictor and AKT in both MEFs and HeLa cells (Fig. 1g and Supplementary Fig. 1g) in a time- and dose-dependent fashion (Supplementary Fig. 1h,i) compared with the control. Furthermore, treatment with octyl-2HG caused a relative increase in cell size (Fig. 1h), consistent with activation of mTOR by 2HG. 2HG-mediated effects on phosphorylation of mTORC1 substrates and cell size were attenuated by rapamycin (Supplementary Fig. 1j,k), and effect on mTORC2 activity inhibited by Torin1 (Supplementary Fig. 1l). Taken together, our results demonstrate that IDH1/2 mutations engender an increase in 2HG levels that stimulate mTOR signalling via both mTORC1 and mTORC2.

### KDM4A modulates mTOR activity

**Figure 2:**
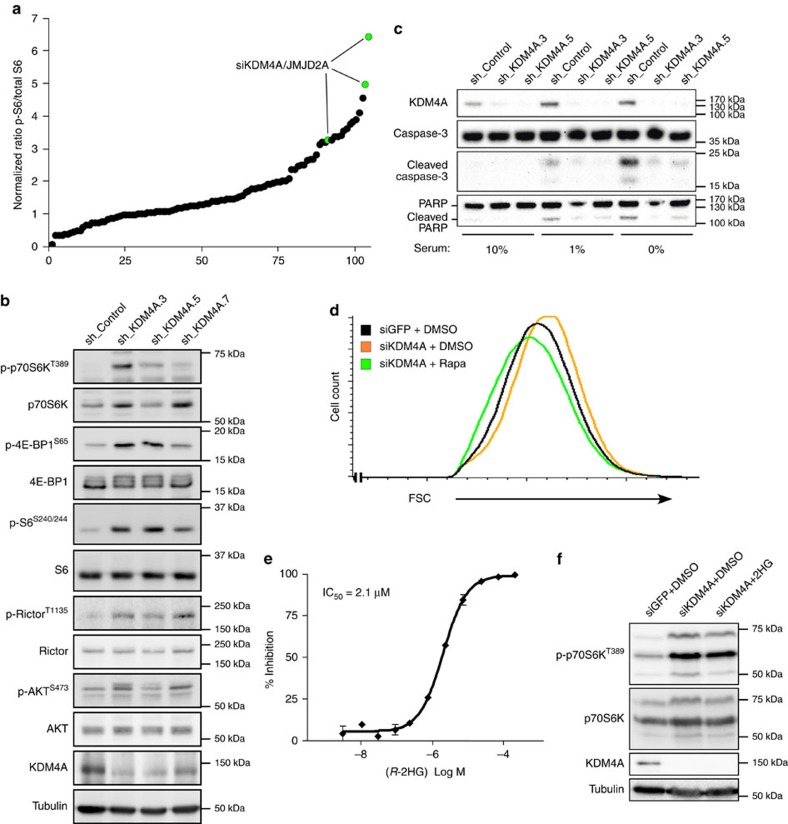
Targeted-siRNA screen reveals KDM4A as an α-KG-dependent enzyme modulating mTOR activity. (**a**) siRNA screen of αKG-dependent enzymes for the involvement in mTOR signalling in HeLa cells. The experiments were performed in triplicate and the results of all experiments were plotted. The levels of phosphorylated S6 on serines 240/244 were normalized to total S6 protein. (**b**) KDM4A knockdown increases mTORC1/2 signalling. Depletion of KDM4A by lentiviral infection in HeLa cells using three different shRNAs. Whole-cell lysates were blotted for mTORC1/2 activation. (**c**) Depletion of KDM4A protects against apoptosis induced by serum deprivation. HeLa cells expressing a control shRNA or shRNAs targeting KDM4A were grown in media containing the indicated concentrations of serum for 24 h. Cell lysates were then analysed by western blotting. (**d**) KDM4A depletion increases cell size in an mTORC1-dependent manner. MEFs p53^−/−^ were treated with siKDM4A and, where indicated, with DMSO or 25 nM rapamycin, for 16 h before FSC measurements by FACS. A total 200,000 cells were counted for each condition. (**e**) Dose-dependent inhibition of KDM4A by *R*-2HG in *in vitro* demethylation assay (graph represents two independent experiments). (**f**) 2HG does not further stimulate mTORC1 in KDM4A-depleted HeLa cells. HeLa cells transfected with siGFP or siKDM4A were serum starved for 16 h before stimulation with 2 mM octyl-2HG for 4 h.

Previous studies showed that mutated IDH1/2-stimulated 2HG production inhibits the activity of αKG-dependent enzymes including (i) the Jumonji family of Fe(II)-dependent oxygenases comprising lysine demethylases and (ii) the TET family of 5-methylcytosine hydroxylases^6^,^7^. To evaluate the possibility that one or more enzymes among these families is/are responsible for regulation of mTORC1 activity, we performed a targeted short interfering RNA (siRNA) screen in HeLa cells using S6 phosphorylation on serines 240 and 244 as a readout of mTOR activity. Among the αKG-dependent enzymes tested, depletion of KDM4A showed the most potent increase in S6 phosphorylation (Fig. 2a). KDM4A is an Fe(II)- and αKG-dependent enzyme possessing a JmjC catalytic domain^8^,^9^, as well as tandem PHD- and tandem Tudor- domains which are thought to associate with methylated chromatin residues. KDM4A catalyses demethylation of di- and tri-methylated H3K9 and H3K36 (refs 9, 39, 40, 41), and modulates multiple cellular processes including the DNA damage response^13^, cellular senescence^14^,^42^,^43^, transcription^40^,^44^, DNA replication^12^,^45^ and innate immunity^46^. Knockdown of KDM4A using three different short hairpin RNAs (shRNAs) led to increased phosphorylation of the mTORC1 substrates p70S6K^T389^ and 4E-BP1^S65^, and of the mTORC2 substrate AKT^S473^ (Fig. 2b), thus validating our siRNA screening approach. As AKT modulates cell survival and depletion of KDM4A leads to AKT activation (Fig. 2b), we determined the function of KDM4A in cell survival on metabolic stress. KDM4A knockdown prevented cleavage of caspase-3 and PARP, two indicators of apoptosis, following serum withdrawal (Fig. 2c). Moreover KDM4A-depleted cells were larger than cells treated with siGFP, and treatment with the mTORC1 inhibitor rapamycin abrogated this phenotype (Fig. 2d). We emphasize that the entire KDM4 family shares common substrates; nonetheless KDM4A was the sole family member to influence mTORC1 activity, as depletion of KDM4B-D had no impact on phosphorylation of 4E-BP1, S6 and Rictor (Supplementary Fig. 2a,b). KDM4A is profoundly inhibited by 2HG, exhibiting an IC_50_ of 2.1 μM (Fig. 2e) as determined by RapidFire mass spectrometry. 2HG failed to further increase mTORC1 activity in KDM4A-depleted cells (Fig. 2f), suggesting that inhibition of KDM4A by 2HG-producing mutated IDH1/2 is responsible for mTORC1/2 activation.

### KDM4A interacts with mTORC1/2

**Figure 3:**
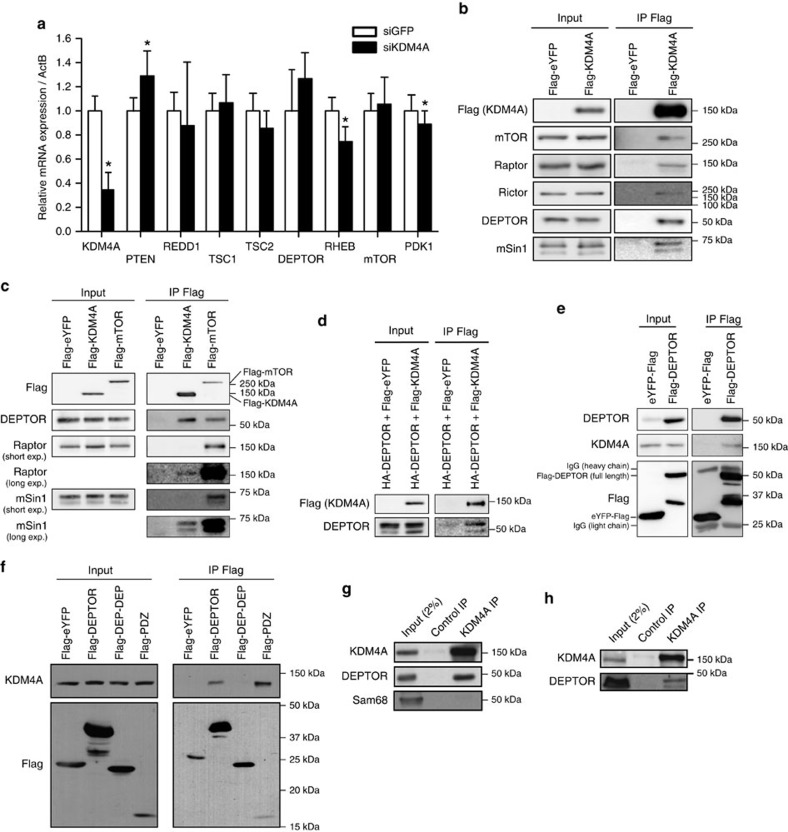
KDM4A interacts with the mTORC1/2 complex. (**a**) Relative mRNA levels of negative and positive regulators of the PTEN/AKT/mTOR pathway following KDM4A depletion. Quantifications of mRNAs by RT–qPCR were normalized against β-actin (*ActB*) mRNA. Asterisks denote a statistical difference between siKDM4A-treated cells and siGFP control cells, two-sided *t*-test *P*<0.05 (graph represents two independent experiences). Error bars represent standard deviation. (**b**) Co-immunoprecipitation of endogenous mTORC1/2 complex members with Flag-KDM4A in 293T transfected cells. (**c**) Comparison of mTORC1/2-associated proteins with Flag-tagged mTOR or KDM4A. The 293T cells were transfected with either Flag-eYFP, Flag-KDM4A or Flag-mTOR, and protein lysates were subjected to anti-Flag immunoprecipitation. (**d**) Co-immunoprecipitation of Flag-KDM4A and HA-DEPTOR in 293T cells. (**e**) Endogenous KDM4A co-immunoprecipitates with Flag-DEPTOR. (**f**) DEPTOR PDZ domain associates with endogenous KDM4A. Flag immunoprecipitation of flag-tagged full length or fragments of DEPTOR. The samples were not sonicated in this experiment to confirm that the interaction is independent of nucleus disruption. (**g**) Endogenous KDM4A and DEPTOR associate in 293E cells. (**h**) Endogenous KDM4A and DEPTOR co-immunoprecipitate in NHA-hTERT cells.

To elucidate the molecular mechanism involved in the regulation of mTOR kinase activity by KDM4A, we evaluated whether the latter transcriptionally modulates either PTEN/AKT/mTOR pathway regulators or mTORC1/2 complex components. Depletion of KDM4A in HeLa cells revealed minor or no significant changes in mRNA levels for either PTEN or tuberous sclerosis 1/2 (TSC1/2) (Fig. 3a). TSC1/2 is a GTPase-activating protein complex that maintains Rheb (Ras homologue enriched in brain) in an inactive form, thereby causing mTOR inhibition^47^,^48^,^49^. In addition, mRNA levels of RHEB, mTOR, DEPTOR or the upstream activating kinase PDK1 were at most very modestly changed following KDM4A depletion (Fig. 3a). Similar observations were made at the protein level for KDM4A-depleted cells, where expression of PTEN, TSC2, mTOR, Rictor and components of the amino acid signalling arm (that is, Rag and LAMTOR) remained essentially unaltered (Supplementary Fig. 2c,d). Interestingly, KDM4A knockdown had no impact on phosphorylation of AKT at threonine 308, a site phosphorylated by PDK1 after PI3K activation, suggesting that activation of mTORC1 occurs downstream PDK1/AKT (Supplementary Fig. 2c). This was further confirmed by our result showing that pharmacological PI3K inhibition using CAL-101 did not block activation of mTORC2 upon depletion of KDM4A, as revealed by increased phosphorylation of AKT^S473^ (Supplementary Fig. 2e). Furthermore, knockdown of KDM4A in rapamycin-treated cells still caused activation of mTORC2, implying mTORC1-independent modulation of mTORC2 by KDM4A (Supplementary Fig. 2e).

Since the control exerted by KDM4A on mTOR activity seems to occur downstream of PTEN–PDK1–AKT–TSC1/2, we investigated whether KDM4A might physically interact with the mTORC1/2 complexes. Consistent with this possibility, KDM4A co-immunoprecipitated numerous members of mTORC1 and mTORC2 including mTOR, Raptor, Rictor, mSin1 and DEPTOR (Fig. 3b and Supplementary Fig. 3a–c). We then qualitatively compared the amount of mTORC1/2 components interacting with mTOR or KDM4A. Flag-tagged KDM4A modestly interacted with mSin1 and Raptor when compared with the amount co-immunoprecipitated with mTOR. On the other hand, both KDM4A and mTOR immunoprecipitated similar amounts of DEPTOR (Fig. 3c and Supplementary Fig. 3d), suggesting that DEPTOR might represent the major mTORC1/2 component interacting with KDM4A. We confirmed the interaction between DEPTOR and KDM4A in cells co-expressing the exogenous tagged proteins (Fig. 3d). Moreover, ectopic Flag-tagged DEPTOR co-immunoprecipitated endogenous KDM4A (Fig. 3e) and this interaction appeared to be mediated by the DEPTOR PDZ domain (Fig. 3f). Individual depletion of mTOR or mSin1 did not alter the binding of KDM4A and DEPTOR (Supplementary Fig. 3e,f), supporting our hypothesis that DEPTOR is the major mTORC1/2 component interacting with KDM4A. Endogenous KDM4A and DEPTOR are also associated in 293E (Fig. 3g), HeLa (Supplementary Fig. 3g), as well as in glioma-related central nervous system-derived cells, that is, immortalized normal human astrocytes (NHAs; Fig. 3h). Surprisingly, endogenous KDM4B also interacted with DEPTOR (Supplementary Fig. 3h), but was unable to modulate mTOR activity (Supplementary Fig. 2a). Recombinant KDM4A and DEPTOR proteins purified from *E.coli* failed to interact (Supplementary Fig. 3i), suggesting that KDM4A–DEPTOR interaction might be indirect, or requires either post-translational modifications absent in bacteria or conformational changes in DEPTOR occuring during its association with mTORC1 or mTORC2. We performed a fractionation assay to confirm that KDM4A and DEPTOR localize to the same sub-cellular compartments. While DEPTOR is mostly cytoplasmic, KDM4A was present in both the nucleus and cytoplasm (Supplementary Fig. 4a,b).

### KDM4A stabilizes DEPTOR

**Figure 4:**
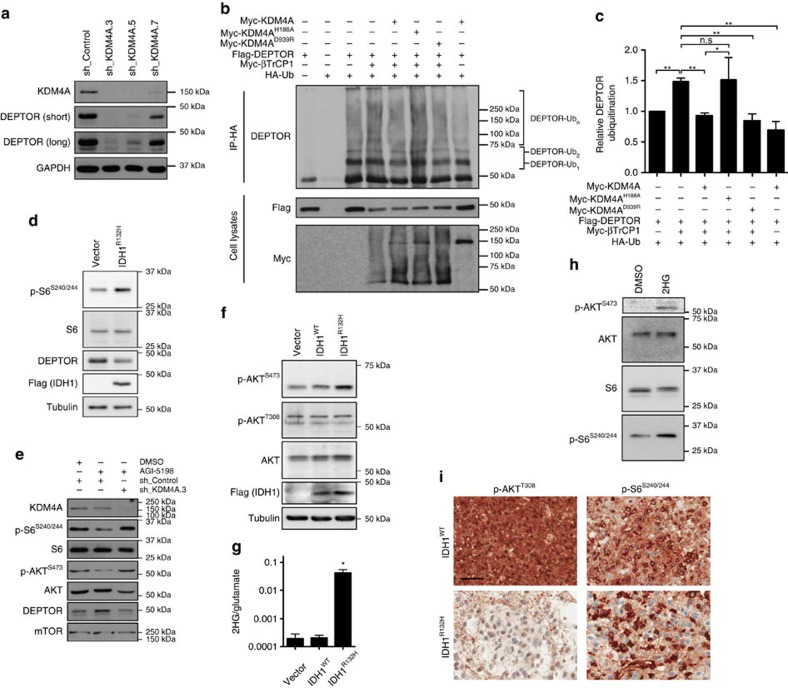
Functional implication of IDH1/2 mutations in central neuron system-derived cells. (**a**) KDM4A depletion reduces DEPTOR levels. HeLa cells were transduced with lentivirus expressing sh_KDM4A.3, sh_KDM4A.5, sh_KDM4A.7 or sh_Control and whole-cell lysates were blotted to determine DEPTOR levels. (**b**) The demethylase activity of KDM4A is required to block β-TrCP1-dependent ubiquitination of DEPTOR *in vivo*. HA IP was performed on 293E cells transfected with the indicated plasmids. (**c**) Quantification of DEPTOR ubiquitination by β-TrCP1 in the presence or absence of wild-type KDM4A or mutants. DEPTOR ubiquitination was quantified in three independent experiments using ImageJ. Asterisks denote a statistical difference, two-sided *t*-test, *P<0.05; **P<0.01. Error bars represent standard deviation. NS, not significant, *P*>0.05. (**d**) IDH1^R132H^ expression diminishes DEPTOR levels. Flag-IDH1^R132H^ was introduced in p53^−/−^ MEFs by retroviral transduction and whole-cell lysates immunoblotted. (**e**) Inhibition of endogenous mutated IDH1^R132C^ decreases mTOR activity in a KDM4A-dependent manner. HT1080 cells were transduced with lentivirus expressing sh_KDM4A.3 or sh_Control. The cells were treated with 3 μM AGI-5198 twice every day for 5 days and whole-cell lysates blotted for markers of mTOR activation. (**f**) Expression of mutant IDH1 in NHA-hTERT increases mTOR signalling. IDH1^WT^-Flag or IDH1^R132H^ genes were introduced in astrocytes through retroviral infection and cell lysates were blotted for mTORC2 activation. (**g**) IDH1^R132H^ overexpression increases 2HG production in astrocytes. Intracellular 2HG levels were normalized to glutamate. Asterisk denotes a statistical difference between IDH1^R132H^ and empty vector cells, two-sided *t*-test *P*<0.0001 (graph represents three independent experiments). Error bars represent standard deviation. (**h**) Treatment of NHA-hTERT with octyl-2HG activates mTOR signalling. The cells were treated for 8 h with 1 mM octyl-2HG. The experiment shown is a representative of triplicates. (**i**) Non-canonical mTOR activation in IDH1/2^WT^ and IDH1^R132H^ human astrocytomas. Scale bar, 50 μm. Representative samples from IDH1/2^WT^ (*n*=3) and IDH1^R132H^ (*n*=3) human astrocytomas are shown.

The stability of DEPTOR, an endogenous negative regulator of mTOR activity^24^, is tightly regulated in response to growth signals via mTOR in conjunction with the SCF(βTrCP) E3 ubiquitin ligase complex^26^,^27^,^28^. We sought to determine the impact of KDM4A on DEPTOR protein levels. KDM4A knockdown led to a significant decrease in both DEPTOR protein levels (Fig. 4a) and half-life after serum stimulation (Supplementary Fig. 5a-b). Transient expression of wild-type KDM4A or tandem Tudor-disrupted KDM4A^D939R^, but not of catalytically inactive KDM4A^H188A^, in KDM4A-depleted cells partially restored DEPTOR levels (Supplementary Fig. 5c,d). This partial rescue might reflect reduced transfection efficiency (40–50%) of lentiviral-transduced cells under our experimental conditions. It is also possible that the transient nature of KDM4A re-introduction may not allow enough time for complete restoration of DEPTOR levels. We cannot exclude, however, that a portion of the effects we observed on DEPTOR levels are independent of KDM4A and maybe due to shRNA off-targeting effects. In addition, overexpression of wild-type KDM4A significantly abrogated *in vivo* ubiquitination of DEPTOR by β−TrCP1 (Fig. 4b,c); moreover, this reduction was dependent on the catalytic activity of the demethylase, but not on its chromatin-binding ability, since overexpression of the KDM4A^D939R^ Tudor domain mutant, but not of the KDM4A^H188A^ catalytic dead mutant, decreased DEPTOR poly-ubiquitination (Fig. 4b,c). In addition, KDM4A interacted with β-TrCP ubiquitin ligases (Supplementary Fig. 5e). Consistent with our finding that inhibition of KDM4A decreased DEPTOR protein levels, expression of the 2HG-producing IDH1^R132H^ mutant also caused such a reduction (Fig. 4d). Treatment of human fibrosarcoma HT1080 cells bearing a heterozygous IDH1^R132C^ mutation with the mutated-IDH1-specific inhibitor AGI-5198 (ref. 37) caused a marked decrease in mTORC1/2 activity. Moreover this was rescued upon KDM4A depletion (Fig. 4e and Supplementary Fig. 5f,g), showing that 2HG-mediated inhibition of KDM4A is indeed responsible for activation of mTOR. Our data raise the intriguing possibility that cancer therapeutic regimens designed to inhibit mutated IDH1/2 would be expected to reduce oncogenic mTOR activity.

### IDH1 mutation and 2HG regulate mTOR in human astrocytes

To evaluate the impact of IDH mutations on mTOR activation in a more physiological setting, we first retrovirally transduced immortalized NHAs with empty vector, wild-type IDH1 or IDH1^R132H^. NHAs expressing mutant IDH1 displayed higher levels of p-AKT^S473^ (Fig. 4f) associated with increased accumulation of intracellular 2HG (Fig. 4g), but did not manifest decreased αKG (Supplementary Fig. 5h). In addition, treatment of NHAs with octyl-2HG led to activation of mTORC1 and mTORC2, as indicated by increased p-S6^S240/244^ and p-AKT^S473^, respectively (Fig. 4h). Finally, octyl-2HG decreased DEPTOR levels in NHA, as compared with control (Supplementary Fig. 5i).

**Figure 5:**
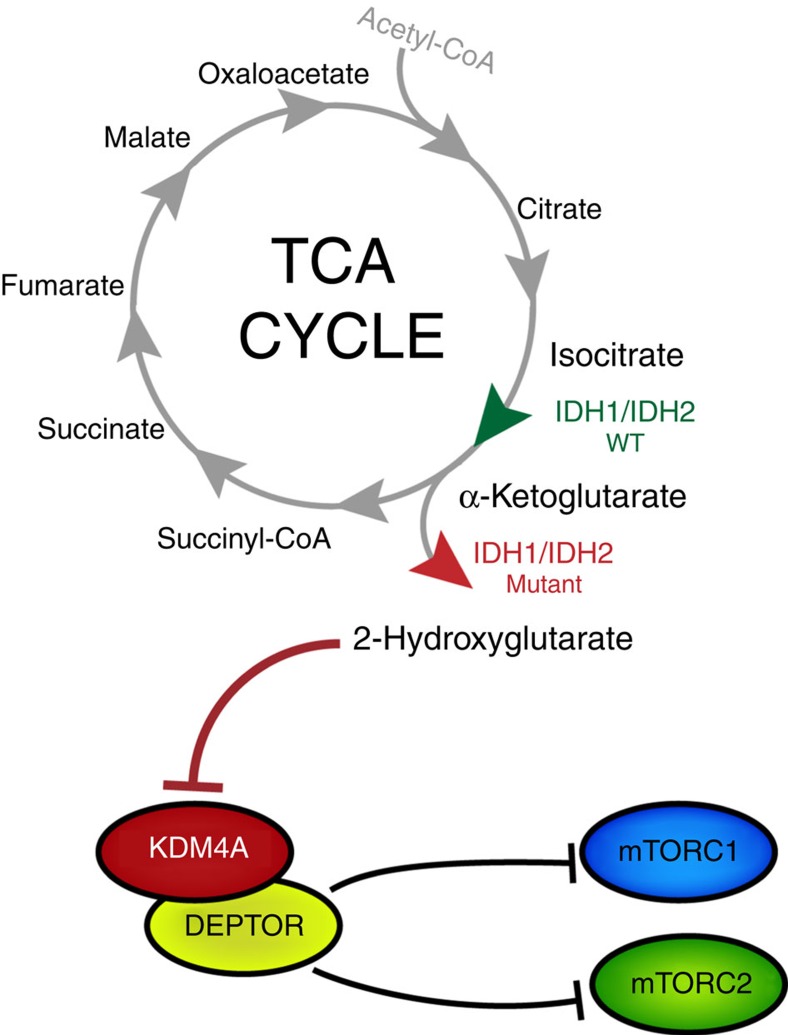
Non-canonical regulation of mTOR activity by 2HG-mediated inhibition of KDM4A. Simplified schematic model for mTOR activation following KDM4A inhibition by *IDH1/2* gain-of-function mutations.

We then asked whether oncogenic IDH1 might be associated with markers of mTOR activation in human brain tumours. Immunohistochemistry analysis of IDH1^R132H^-mutated human-grade 2/3 astrocytoma samples revealed an increase in S6 phosphorylation on serines 240/244 (Fig. 4i). However, while 20 to 40% of the tumour cells from wild-type IDH1/2 astrocytomas showed strong staining for p-AKT^T308^, the upstream activator of mTORC1, less than 2% of IDH1^R132H^-mutated astrocytomas stained positively for p-AKT^T308^ (Fig. 4i). This suggests that activation of mTORC1 occurs downstream of the PI3K–PDK1–AKT cascade in tumours harbouring IDH1 mutations, supporting the notion that 2HG-mediated inhibition of KDM4A affects the stability of DEPTOR leading to mTOR activation independently of the PI3K/AKT/TSC1-2 pathway (Fig. 5).

## Discussion

IDH mutations, frequently observed in brain tumours and leukaemia, lead to production of the oncometabolite 2HG, which in turn causes cell differentiation defects by impairing histone demethylation^38^. In addition, reversible inhibition of the 5′-methylcytosine hydroxylase TET2 by 2HG causes profound epigenetic changes contributing to transformation^50^,^51^. Herein we describe for the first time that oncogenic IDH and subsequent production of 2HG activate mTOR signalling. Either expression of mutated IDH1/2 or treatment with 2HG engendered activation of both mTORC1 and mTORC2 and, as a consequence, cell size increase. We note that the mTORC1 inhibitor rapamycin significantly, but only partially, decreased cell size of IDH2^R172K^-expressing cells. In mammalian systems, control of cell size by mTOR is mediated by both p70S6K and 4E-BP/eIF4E^36^. As these latter factors display differential sensitivity to rapamycin^52^, recovery of 4E-BP1 phosphorylation in rapamycin-treated IDH2^R172K^ cells might explain the observed partial rescue of cell size (Fig. 1e).

Since 2HG constitutes an inhibitor of α-ketoglutarate-dependent enzymes, we used a targeted siRNA screen against these enzymes to identify KDM4A as a novel regulator of mTOR. Furthermore, our data indicate that KDM4A controls mTOR through modulation of DEPTOR stability. Indeed, KDM4A catalytic activity is essential for decreasing β-TrCP1-dependent poly-ubiquitination of DEPTOR (Fig. 4b,c) thereby promoting DEPTOR protein stability (Supplementary Fig. 5c). Further investigation will be required to identify the substrate(s) of KDM4A-mediated demethylation involved in controlling DEPTOR stability.

Amplification of *KDM4A* gene copy number, or of its mRNA expression, has been observed in various cancers. Such upregulation of KDM4A has been reported to promote site-specific genomic amplification^45^ or hinder p53 activation and senescence^14^. Conversely, genomic loss of *KMD4A* and concomitant decrease in mRNA levels has also been reported in multiple tumour types (Supplementary Fig. 6a–j)^45^, particularly bladder cancer where KDM4A downregulation correlates with tumour progression^53^. Our own results suggest that decreased KDM4A expression could contribute to mTOR activation during cancer development, suggesting that this demethylase could act as a context-dependent oncogene.

Recent evidence demonstrates that mTORC1 controls mitochondrial activity^54^ and stimulates metabolism of glutamine^55^, an amino acid catabolized into αKG, suggesting that mTOR modulates the TCA cycle. Here we provide evidence that TCA cycle defects can lead to aberrant activation of mTOR kinase. Indeed our data show that KDM4A associates with DEPTOR, and that 2HG-mediated inhibition of KDM4A in IDH1/2-mutated cells impacts DEPTOR protein stability leading to mTOR activation (Fig. 5). On the other hand, it has been reported that 2HG inhibits ATP synthase and mTOR activity in cell lines wherein mTOR is chronically hyperactivated due to the loss of PTEN activity or other causes^56^. Under such conditions, 2HG-mediated inhibition of ATP synthase might alter the ATP/ADP ratio leading to a decrease in mTOR activity. As a possible explanation for this controversy, we emphasize that our genomic analysis of human gliomas and glioblastomas revealed that *IDH* and *PTEN* mutations are mutually exclusive. In addition, our data show that 2HG stimulates mTORC1/2 activity in numerous cell lines where mTOR is not hyperactivated by abrogation of PTEN. Furthermore, mTOR signalling is readily observed in clinical samples of IDH1-mutated gliomas. This clearly suggests that the effects of 2HG on mTOR signalling rely on the integrity of upstream signalling pathways.

The novel PTEN-independent mode of mTORC1/2 activation via mutated IDH/2HG/KDM4A/DEPTOR revealed here may provide an additional molecular explanation for the oncogenic activity of IDH1/2 mutation in brain cancer. This would indicate a potential therapeutic strategy to target oncogenic mTOR signalling in cancers harbouring IDH1/2 mutations.

## Methods

### Cell culture

HEK293T, HeLa, HT1080 and 293E were obtained from the American Type Culture Collection (ATCC), p53^−/−^ MEFs were obtained from Dr Gerardo Ferbeyre and previously described^57^, and normal human astrocytes immortalized by hTERT (NHA-hTERT) were kindly provided by Dr Nada Jabado. All the cell lines were cultured in Dulbecco's modified Eagle's medium (DMEM, Gibco) supplemented with 10% fetal bovine serum (FBS, Hyclone), 1% sodium pyruvate (Gibco) and 1% penicillin/streptomycin (Gibco). Mycoplasma contamination check has been carried out for all cancer cell lines.

### Plasmids and transfections

Cells were transfected with 10 μg of DNA using 30 μg of linear polyethylenimine. The following vectors were used: pcDNA3.1 Flag-eYFP, pLPC Flag-KDM4A, pCMV-Sport6-Flag-mTOR, pMSCV HA-DEPTOR, pDEST myc-KDM4A wild type or mutants (H188A and D939R), pcDNA3 Myc-β-TrCP1 (Addgene plasmid #20718; gift from Dr Yue Xiong) and pcDNA3 HA-Ub. The following plasmids, pRK5 Myc-mTOR (Addgene plasmid #1861), pRK5 Myc-Raptor (Addgene plasmid #1859), pRK5 Myc-Rictor (Addgene plasmid #11367), pRK5 Flag-DEPTOR (Addgene plasmid #21334), pRK5 Flag-DEPTOR DEP domains (Addgene plasmid #21700) and pRK5 Flag-DEPTOR PDZ domain (Addgene plasmid #21701) were gifts from Dr David Sabatini.

### Lentiviral and retroviral infections

Lentiviral particles were generated by transfecting 293T cells using 5 μg of shRNA plasmid, 3.75 μg of psPAX2 packaging plasmid and 1.25 μg pMD2.G envelope plasmid. Media was changed 24 h after transfection and lentiviral particles were harvested 24 h later. Viral supernatant was filtered through 0.45 μm filters and supplemented with 8 μg ml^−1^ polybrene and 10% FBS. Viruses were titrated using limiting dilution in HeLa cells and the same MOI was used for each condition. The following plasmids were used: pLKO.1-sh_Control, pLKO.1-sh_KDM4A#3 (RHS3979-201745071, Dharmacon), pLKO.1-sh_KDM4A#5 (RHS3979-201745073, Dharmacon), pLKO.1-sh_KDM4A#7 (RHS3979-201745075, Dharmacon), pLKO.1-sh_mTOR#53 (TRCN0000195453, Sigma), pLKO.1-sh_mTOR#84 (TRCN0000039784, Sigma), pLKO.1-sh_mSin1#1 (13483, Addgene) and pLKO.1-sh_mSin1#2 (13484, Addgene).

Retroviruses were produced by transfecting packaging cells (Phoenix) with 10 μg of DNA using 30 μg of polyethylenimine. Media was changed 24 h later and viral supernatant harvested at 48 and 56 h post-transfection. Viral supernatants were filtered through 0.45 μm filters and supplemented with 8 μg ml^−1^ polybrene and 10% FBS. Media were changed 16 h after infection. Retroviral plasmids pLPCX IDH1^WT^ Flag, pLPCX IDH1^R132H^ Flag, pLPCX IDH2^WT^ Flag and pLPCX IDH2^R172K^ Flag are described in ref. 58. The pLXSN IDH1^WT^ Flag and pLXSN IDH1^R132H^ Flag were generated by PCR of the IDH1 genes (forward primer with EcoR1 site : 5′-TAGAATTCATGTCCAAAAAAATCAGTGG-3′, reverse primer with Xho1 site and Flag tag : 5′-ACCTCGAGTTACTTGTCATCGTCATCCTTGTAATCCATAAGTTTGGCCTG-3′). PCR products were inserted into the EcoRI- and XhoI-linearized pLXSN vector.

### FACS-cell size determination

The cells were trypsinized and resuspended in phosphate-buffered saline (PBS) containing 2% FBS. Forward-scatter measurement (FSC) intensity was measured using BD FACSCalibur (BD Biosciences) and analysed with BD CellQuest Pro software (BD Biosciences). For each experiment 1,00,000–2,00,000 cells were analysed, except for cells treated with 2HG where 10,000–25,000 cells were used.

### Immunoblots and antibodies

The cells were lysed in 10 mM Tris (pH 7.4), 120 mM NaCl, 0.5% Triton with protease inhibitor mix (cOmplete, Roche) and phosphatase inhibitors (PhosSTOP, Roche) and sonicated for 3 × 10 s. After centrifugation at 15,000*g* for 10 min, proteins were separated on SDS–PAGE and transferred to nitrocellulose or PVDF membranes (Bio-Rad). Primary antibodies used were anti-Flag (M2, Sigma, 1:5,000), anti-HA.11 (16B12, Covance, 1:1,000), anti-Myc (9E10, Sigma, 1:1,000), anti-α-Tubulin (B-5-1-2, Sigma, 1:10,000), anti-KDM4A (NB110-40585, Novus Biologicals, 1:1,000) and (N154/32, NeuroMab, 1:1,000), anti-mTOR (2972, Cell Signaling, 1:1,000) (7C10, Cell Signaling, 1:1,000), anti-DEPTOR (ABS222, Millipore, 1:1,000), anti-DEPTOR (D9F5, Cell Signaling, 1:500), anti-DEPTOR (NBP1-49674, Novus Biologicals, 1:1,000), anti-GβL (PA5-17442, Thermo Scientific, 1:1,000), anti-Raptor (24C12, Cell Signaling, 1:1,000), anti-phospho-Raptor S792 (2083, Cell Signaling, 1:1,000), anti-PRAS40 (PA5-17184, Thermo Scientific, 1:1,000), anti-Rictor (53A2, Cell Signaling, 1:1,000), anti-phospho-Rictor^T1135^ (D30A3, Cell Signaling, 1:1,000), anti-mSin1 (07-2276, Millipore, 1:1,000), anti-Protor2 (D19A5, Cell Signaling, 1:1,000), anti-PTEN (A2B1, Santa Cruz, 1:1,000), anti-TSC2 (T.309-0 Thermo Scientific, 1:1,000), anti-RagA (D8B5, Cell Signaling, 1:1,000), anti-RagB (D18F3, Cell Signaling, 1:1,000), anti-RagC (D31G9, Cell Signaling, 1:1,000), anti-RagD (4470, Cell Signaling, 1:1,000), anti-LAMTOR1 (D11H6, Cell Signaling, 1:1,000), anti-LAMTOR2 (D7C10, Cell Signaling, 1:1,000), anti-LAMTOR3 (D38G5, Cell Signaling, 1:1,000), anti-p70S6K (9202, Cell Signaling, 1:1,000), anti-phospho-p70S6K^T389^ (108D2, Cell Signaling, 1:1,000), anti-4E-BP1 (53H11, Cell Signaling, 1:1,000), anti-phospho-4E-BP1^S65^ (9451, Cell Signaling, 1:1,000), anti-S6 (54D2, Cell Signaling, 1:1,000), anti-phospho-S6^S240/244^ (5364 and 2215, Cell Signaling, 1:1,000), anti-AKT PH domain (05-591, Millipore, 1:1,000), anti-phospho-AKT^S473^ (9271, Cell Signaling, 1:1,000) (D9E, Cell Signaling, 1:1,000), anti-phospho-AKT^T308^ (C31E5E, Cell Signaling, 1:1,000), anti-PARP (9542, Cell Signaling, 1:1,000), anti-Caspase 3 (9662, Cell Signaling, 1:1,000), anti-actin (D6A8, Cell Signaling, 1:2,000) and anti-GAPDH (D16H11, Cell Signaling, 1:2,000). Scans of ECL and/or X-ray films showing whole membranes are presented in Supplementary Fig. 7.

### Immunoprecipitations

The cells were lysed in 1 ml of lysis buffer (0.3% CHAPS, 50 mM Hepes, 120 mM NaCl, 2 mM EDTA with protease inhibitor mix and phosphatase inhibitors (Roche)) and sonicated three times for 10 s each (except for Fig. 3f, for which samples were not sonicated to confirm that the interaction between KDM4A and DEPTOR occurred prior sonication). After centrifugation at 15,000*g* for 10 min, the supernatant was incubated for 2 h at 4 °C with 20 μl of Flag M2 agarose beads (Sigma). Immunoprecipitates were washed three times with lysis buffer containing 150 mM NaCl and twice with TBS before elution in Laemmli buffer.

For qualitative comparison of IP performed in CHAPS and Triton (Supplementary Fig. 3d), the cells were lysed in lysis buffer (40 mM Hepes pH 7.5, 120 mM NaCl, 1 mM EDTA with protease inhibitor mix Roche) containing either 0.25% CHAPS or 1% triton. The cells were then sonicated three times for 10 s each before centrifugation at 15,000*g* for 10 min. The supernatants were incubated for 1 h at 4 °C with 20 μl of Flag M2 agarose beads (Sigma). The immunoprecipitates were washed five times with lysis buffer and eluted in Laemmli buffer.

For HA-Ub IP, the cells were serum starved for 24 h followed by 30 min serum stimulation (10%) before addition of lysis buffer A (20 mM Tris pH 7.5, 1% Triton, 150 mM NaCl, 1 mM EDTA, 1 mM EGTA, 20 mM *N*-ethylmaleimide with protease inhibitor mix and phosphatase inhibitors (Roche)). Sample were sonicated three times for 10 s each and immunoprecipitated with 20 μl of HA agarose beads (Sigma) for 2 h at 4 °C. The immunoprecipitates were washed three times with lysis buffer A before elution in Laemmli buffer.

For endogenous KDM4A IP, ∼2 × 10^8^ cells (NHAs, HeLa or 293E) were resuspended in 5 ml of lysis buffer A with 0,5% triton. Extracts were prepared as described above and were pre-cleared for 1 h with 20 μl protA/G beads (Cell Signaling). Immunoprecipitations were performed with 12 μg of NO66 antibody (negative control, SCG consortium, ABC058-F03), KDM4A antibody (p014, SCG consortium) or KDM4B antibody (SCG consortium) overnight at 4 °C. 60 μl of M-280 streptavidin-coated dynabeads (Life Technologies) were added to each sample and incubated for 1 h at 4 °C. The beads were washed three times with lysis buffer A before elution in Laemmli buffer.

### Production of recombinant proteins

The GST-DEPTOR (pGEX 5X3), His-KDM4A(aa 1-562) and His-KDM4A(aa 563-1064) (pDest17) were expressed in BL21 bacteria grown in LB. After induction with IPTG (0.2 mM) for 16 h at 20 °C, bacteria were harvested by centrifugation and lysed by sonication. The GST-fusion protein was purified on glutathione agarose beads (Sigma) and the His-fusion protein were purified with Ni-NTA agarose (Qiagen). The His-fusion proteins were eluted off the beads using 200 mM imidazole buffer and dialysed overnight against PBS, followed by concentration on Amicon columns (Millipore).

### GST pull-down assays

For GST pull-downs, 1 μg of purified GST-DEPTOR recombinant protein was incubated for 2 h at 4 °C with 500 ng of His-KDM4A (aa 1–562) or (aa 563–1064) recombinant protein in GST pull-down buffer (25 mM HEPES pH7.5, 250 mM KCl, 0,1% NP-40, 10% glycerol, 100 μg ml^−1^ BSA). The beads were then washed with GST pull-down buffer four times and eluted in Laemmli buffer 2 × .

### Metabolite extraction and quantification of 2HG

Quantification of citric acid cycle (CAC) organic acid intermediates was carried out at the Goodman Cancer Research Center metabolomics core facility (McGill University, Montreal, QC, Canada). Following cellular extraction and either keto-acid reduction or methoximation, GC/MS data were collected and analysed as described in detail^59^,^60^ except that 4 μl of the deuterated CAC standard mix was added due to the high level of 2-hydroxyglutaric acid in mutant IDH1/2-expressing cells. Resulting levels of CAC intermediates were normalized against cell number.

### Synthesis of 1-octyl-*D*-2-hydroxyglutarate

Synthesis of 1-octyl-D-2-hydroxyglutarate was performed as previously described^38^. Briefly, commercial *R*(-)-tetrahydro-5-oxofuran-2-carboxylic acid (910 mg, 7 mmol) was dissolved in water (1 ml), cooled to 0 °C and treated with 1 N KOH (14.7 ml, 14.7 mmol). After stirring, the solution was dessicated under reduced pressure, co-evaporated with EtOH and dried. The white salt obtained was partially dissolved in trifluoroactic anhydride (50 ml) and the solution evaporated under reduced pressure. Anhydrous tetrahydrofuran (45 ml) was then used to dry and dissolve the residue, and octanol (2.3 ml, 14.7 mmol) was added to the resulting solution. After stirring overnight, water was added to quench the reaction and the mixture extracted with EtOAc. The extracts were combined, dried over MgSO_4_, concentrated and purified by flash chromatography eluting with Hexanes-EtOAc 3:1 to 1:1 giving 1-octyl-D-2-hydroxyglutarate (70 mg, 7 %) as an oil. ^1^H NMR (CD_3_OD) δ: 4.19 (m, 1H), 4.15 (m, 2H), 2.51–2.36 (m, 2H), 2.12–2.04 (m, 1H), 1.94–1.85 (m, 1H), 1.70–1.63 (m, 2H), 1.40–1.31 (m, 10H), 0.90 (t, 3H, *J*=6.8 Hz); MS (APCI neg) 259.05 [M-H]-.

### siRNA screen targeting αKG-dependent oxygenases

HeLa cells were transfected using Lipofectamine RNAiMAX (Invitrogen) using reverse transfection protocol supplied by the manufacturer. Briefly, 20 nM siRNA duplexes and 10 μl RNAiMAX lipofectamine (Invitrogen) were diluted in 1 ml OPTI-MEM (Invitrogen) in 6 mm plates and incubated for 15 min before adding 600,000 cells per plate. Three days following the transfection, the cells were lysed in 10 mM Tris-HCl, 100 mM NaCl, 0.5% Triton X-100, pH 7.6 with protease inhibitor mix and phosphatase inhibitors (Roche), sonicated three times for 10 s, then centrifuged for 10 min at 10,000*g*. The supernatants were then analysed by western blotting for total S6 or phospho-S6^S240/244^, and quantification of band intensity was performed using ImageJ. All siRNAs were purchased from ThermoScientific (Dharmacon). For a complete list of siRNAs, see Supplementary Table 1.

### Immunofluorescence

Cells were fixed with 4% paraformaldehyde in PBS for 5 min at room temperature and permeabilized with 1% Triton X-100 in PBS for 5 min at room temperature. Fluorescent visualization of KDM4A was performed using pAb anti-KDM4A antibody (1:100, NB110-40585; Novus Biologicals) coupled with an Alexa Fluor 488 Goat Anti-Rabbit IgG (H+L) secondary antibody (catalogue # 4412, Cell Signaling, 1:1,000). Polymerized actin was visualized using CF™568 phalloidin (catalogue # 00044-300U; Biotium). DNA was stained with 4′,6′-diamidino-2-phenylindole (DAPI). Immunofluorescent images were captured using a × 60 lens (1.42 NA, splan APO), mounted on an FV1000 confocal laser scanning microscope driven by FluoView software (Olympus). Image acquisition was performed at room temperature, the brightness/contrast tool in FluoView software (Olympus) was used to process images and montages were made using Photoshop CS6 (Adobe).

### Cell fractionation

The harvested cells were suspended in cold extraction buffer (10 mM Hepes (pH 7,4), 5 mM MgCl_2_, 320 mM sucrose and 1% Triton X-100), agitated for 10 s and incubated on ice for 10 min. The cells were centrifuged at 2,000*g* and the supernatant (cytoplasmic fraction) collected while the pellet (nuclear fraction) was washed two times with 10 mM Hepes (pH 7,4) containing 5 mM MgCl_2_ and 320 mM sucrose.

### Quantitative real-time PCR

Total RNA was isolated using Trizol (Invitrogen) according to the manufacturer's protocol. The High Capacity cDNA Reverse Transcription Kit was used with random primers (Applied Biosystems) to reverse transcribe 2 μg of total RNA in a final volume of 20 μl. GAPDH (glyceraldehyde-3-phosphate dehydrogenase), HPRT1 (hypoxanthine guanine phosphoribosyl transferase) and ACTB (Beta-actin) were used as endogenous controls and their gene expression levels determined using pre-validated Taqman Gene Expression Assays (Applied Biosystems). Quantitative PCR (qPCR) reactions were performed in 384-well plates using 1.5 μl of cDNA samples (5–25 ng), 5 μl of the TaqMan Fast Universal PCR Master Mix (Applied Biosystems), 0.5 μl of the TaqMan Gene Expression Assay (20X) and 3 μl of water in a final volume of 10 μl. The assays used to determine target gene expression levels were designed using the Universal Probe Library from Roche (www.universalprobelibrary.com). qPCR reactions for target genes were performed in 384-well plates using 1.5 μl of cDNA samples (5–25 ng), 5 μl of the TaqMan Fast Universal PCR Master Mix (Applied Biosystems), 2 μM of each primer and 1 μM of UPL probe in a final volume of 10 μl. Amplification levels were detected using the ABI PRISM 7900HT Sequence Detection System (Applied Biosystems) programmed to run at 95 °C for 3 min before starting 40 cycles of 5 s at 95 °C and 30 s at 60 °C. The reactions were run in triplicate and the quantification performed using average Cts values. The ΔΔCT method was used to determine relative quantification of target genes. The primers used for RT–qPCR (qPCR with reverse transcription) are listed in Supplementary Table 2.

### 2-Hydroxyglutarate IC_50_ determination

The potency of 2-hydroxyglutarate for KDM4A inhibition was determined by RapidFire Mass Spectrometry^61^ and as previously described^62^. Briefly, 25 μl of recombinant human KDM4A (400 nM) was pre-incubated with 2-hydroxyglutarate during 15 min before starting the reaction by adding 25 μl of substrate mix (200 μM L-ascorbic acid, 20 μM Fe^2+^, 20 μM H3K9(me3) peptide and 20 μM 2-oxoglutarate). The enzymatic reaction was stopped after 50 min with 5 μl of 10% formic acid, then the reaction mixture was transferred to a RapidFire RF360 sampling robot. The samples were applied onto a C4 Solid Phase Extraction (SPE) cartridge by aspiration under vacuum. Non-volatile buffer components were removed from the C4 SPE by an aqueous wash step (0.1% formic acid in water). Then, the peptide was eluted from the C4 SPE onto an Agilent 6530 Q-TOF by an organic wash step (85% acetonitrile in water, 0.1% formic acid). Peak area data for the substrate and the product methyl marks were extracted from ion chromatograms and integrated using RapidFire Integrator software. The percentage of demethylation was calculated using Microsoft Excel software, and IC_50_ curves were generated by GraphPad Prism.

### Protein stability assay

For half-life measurements, HeLa cells were starved for 24 h and pre-treated with 100 μg ml^−1^ cycloheximide and 40 μg ml^−1^ chloramphenicol 1 h before serum addition (10%). The cells were harvested at indicated time periods and whole cell lysates blotted. The protein levels were normalized to tubulin and quantified using ImageJ.

### Immunohistochemical stainings of astrocytomas

Diffuse astrocytomas with IDH1^R132H^ mutation (*n*=3) or wild-type IDH1/2 (*n*=3) were processed for immunohistochemistry. IDH1/2 wild-type status was confirmed by Sanger sequencing. Four-micrometre thick sections were cut from the selected formalin paraffin embedded specimens and stained using a Benchmark XT immunostainer (Ventana). The Ventana staining protocol included a pretreatment with Cell Conditioner 1 for 40 min, followed by incubation with the primary antibodies at 37 °C for 60 min. All the antibodies were diluted in the Ventana dilution buffer as followed: p-S6^S240/244^ (D68F8, Cell Signaling Technology, 1:1,000); p-AKT1^T308^ (Novus Biologicals, 1:30). Incubation was followed by detection using Optiview DAB IHC detection kit (Ventana).

### Reproducibility of experiments

Representative figures were repeated as follows. Figs 1d,g; 2b,f and 3g,h and Supplementary Fig. 3g were reproduced at least four times. Figs 1b,c; 3b,f and 4a,b,g,i and Supplementary Figs 1b–d,k; 2b,c,e; 3a,c; 4a,b; 5a,c,g,h,i were repeated three times. Figs 1h and 2c,e, Figs 3a,c and 4d–f,h and Supplementary Figs 1a,g–i,l; 2d; 3b,d,i; 5e,f were reproduced twice.

### Data availability

The TCGA data referenced in this study are available in a public repository from the TCGA website (http://cancergenome.nih.gov). The authors declare that all the other data supporting the findings of this study are available within the article and its Supplementary Information files, and from the corresponding authors upon reasonable request.

## Additional information

**How to cite this article:** Carbonneau, M. *et al*. The oncometabolite 2-hydroxyglutarate activates the mTOR signalling pathway. *Nat. Commun.* 7:12700 doi: 10.1038/ncomms12700 (2016).

## Supplementary Material

Supplementary Information Supplementary Figures 1-7, Supplementary Table 1-2 and Supplementary References
