# Supplementary material for: The oncometabolite 2-hydroxyglutarate activates the mTOR signalling pathway
Source: Nat Commun. 2016 Sep 14;7:12700. doi: 10.1038/ncomms12700 (PMC5027283; doi:10.1038/ncomms12700)
Supplement: Supplementary Information — Supplementary Figures 1-7, Supplementary Table 1-2 and Supplementary References [file ncomms12700-s1.pdf]

Supplementary Information

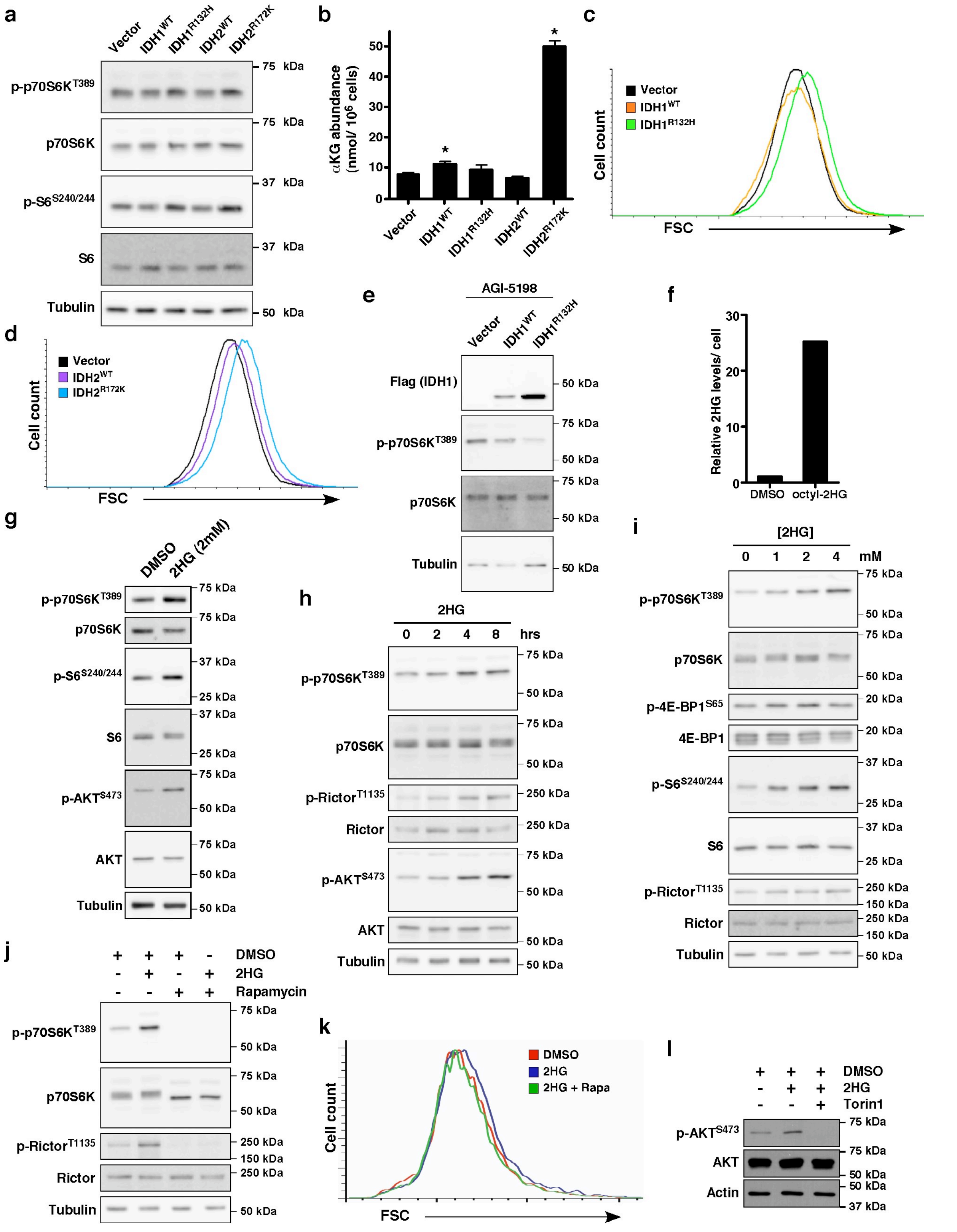

Supplementary Figure 1. 2HG stimulates mTOR kinase. (**a**) mTORC1 activation in MEFs $p53^{-/-}$ cells expressing wt *vs* mutant IDH1/2. Flag-tagged IDH was retrovirally transduced into

MEFs $p53^{-/-}$ cells, and whole cell lysates immunoblotted. (**b**) Metabolomic analysis of αKG levels in MEFs $p53^{-/-}$ cells expressing wt *vs* mutant IDH1/2. Asterisks denote a statistical increase of αKG compared to empty vector control cells, two-sided *t*-test $P < 0.05$ (graph represents n=3 independent experiments). Error bars represent standard deviation. (**c**) Cell size (FSC) of MEFs $p53^{-/-}$ expressing either empty vector (black line), $IDH1^{WT}$ (orange line) or $IDH1^{R132H}$ (green line) was analyzed by FACS. 220 000 cells were counted for each condition and graph is representative of 4 independent experiments. (**d**) Cell size (FSC) of MEFs $p53^{-/-}$ expressing either empty vector (black line), $IDH2^{WT}$ (purple line) or $IDH2^{R172K}$ (blue line) was analyzed by FACS. 220 000 cells were counted for each condition and graph is representative of 4 independent experiments. (**e**) $IDH1^{R132H}$ does not activate mTORC1 in cells treated with AGI-5198, an inhibitor of mutant IDH1. MEFs $p53^{-/-}$ cells were treated twice a day with 10μM AGI-5198 for 5 days, then harvested for western blotting. (**f**) Intracellular accumulation of 2HG in MEFs $p53^{-/-}$ treated with 2mM octyl-2HG. (**g**) HeLa cells were stimulated with 2mM octyl-2HG for 4 hours, and whole cell extracts analysed by Western blot. (**h**) Time course analysis of mTOR activation by 2HG in MEFs $p53^{-/-}$ cells treated with 2mM 2HG freshly added every 4 hours. (**i**) Dose-dependent activation of mTOR by 2HG in MEFs $p53^{-/-}$ cells starved for 24 hours before 2HG addition. (**j**) Rapamycin inhibits 2HG-mediated activation of mTORC1 in MEFs $p53^{-/-}$. Cells were treated with 100nM rapamycin for 24 hours and 2mM 2HG was then freshly added every 4 hours for 8 hours. (**k**) mTORC1 inhibition by rapamycin decreases cell size of MEFs $p53^{-/-}$ treated with 2HG. Cells were pre-treated with 50nM rapamycin, then stimulated with 2mM octyl-2HG for 8 hrs. (**l**) Torin1 treatment prevents mTORC2 activation following stimulation with octyl-2HG. Cells were pre-treated with DMSO or 250 nM Torin1 for 36 hours and then stimulated with 2mM octyl-2HG for 4 hours.

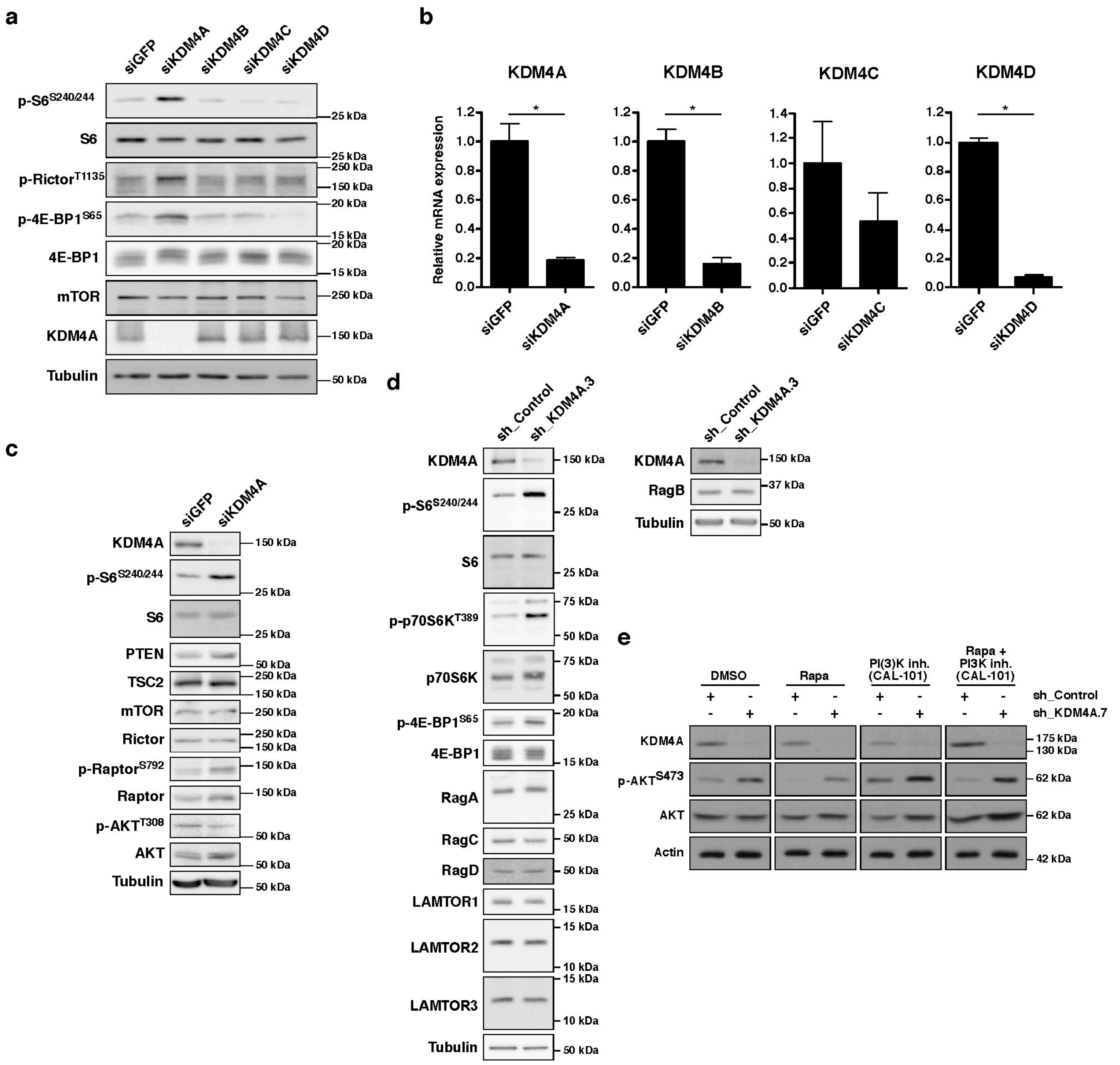

Supplementary Figure 2. KDM4A depletion activates mTOR signaling. (**a**) KDM4A is the only KDM4 family member implicated in mTOR signaling. HeLa cells were treated with siRNAs targeting the KDM4 family members (KDM4A-D) for 72 hours, and lysates blotted for mTOR signaling markers. (**b**) Validation of the KDM4 family members siRNAs by RT-qPCR. Asterisks denote a statistical decrease of KDM4 family member mRNA compared to siGFP control cells, two-sided *t*-test $P < 0.05$ (graph represent technical duplicates and triplicates). Error bars represent standard deviation. (**c**) Protein levels of numerous regulators of mTOR signaling are not modulated by KDM4A. KDM4A was depleted by siRNA transfection in HeLa cells and whole cell lysates were blotted. (**d**) KDM4A depletion does not alter protein levels of regulators of mTOR involved in amino acids sensing. HeLa cells were transduced with lentivirus expressing sh_KDM4A.3 or pLKO.1_Control and whole cell lysate were blotted. (**e**) HeLa cells infected with a sh_Control or sh_KDM4A.7 were serum-starved for 24 hours, then pre-treated or not with 20 nM rapamycin, 1μM of the PI(3)K inhibitor CAL-101 or both for 3 hours, followed by stimulation with serum for 30 minutes.

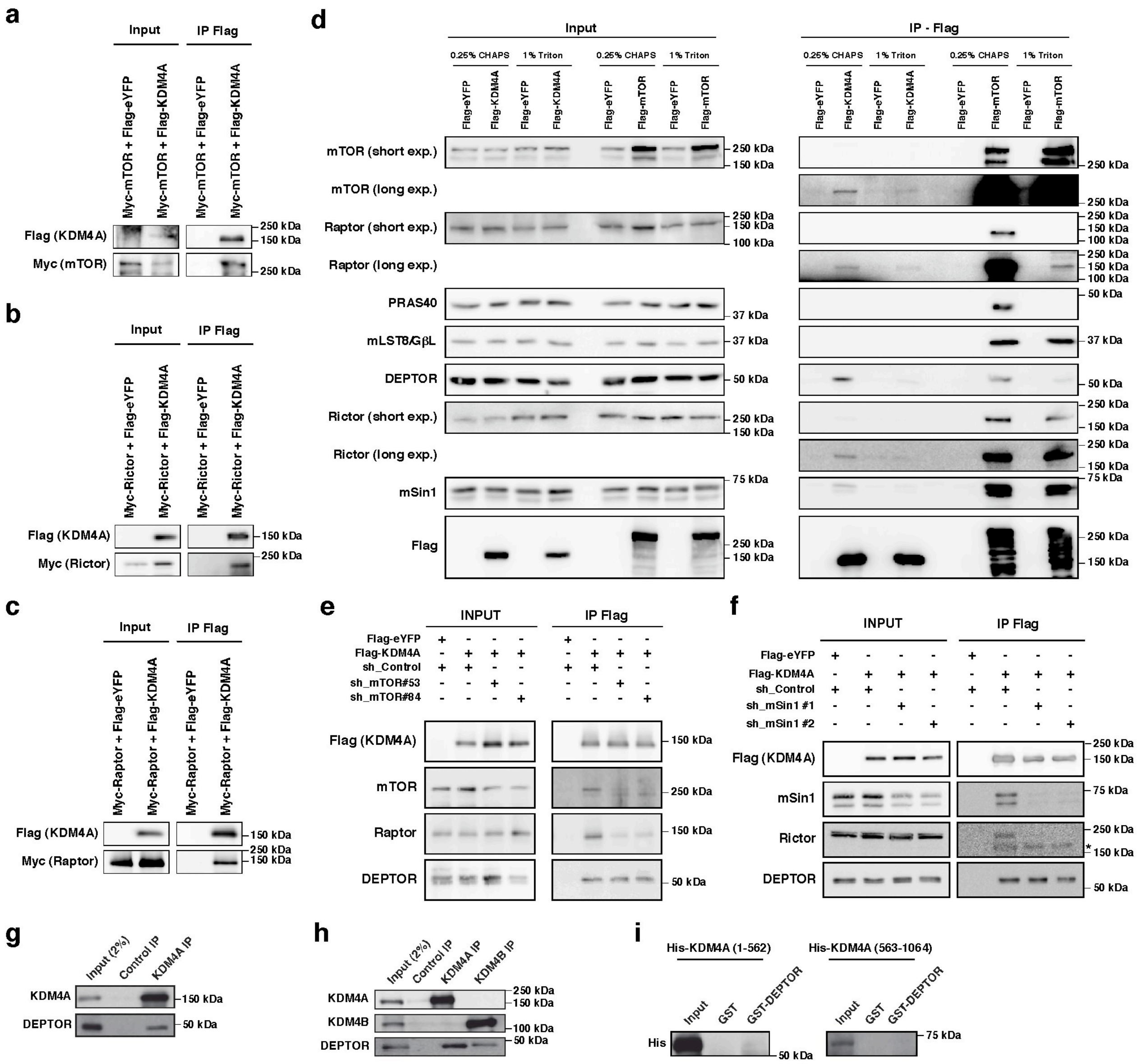

Supplementary Figure 3. Interaction between DEPTOR and KDM4A does not require mTOR or mSin1. (a-c) Co-expression of Flag-KDM4A with Myc-mTOR (**a**), Myc-Rictor (**b**) or Myc-Raptor (**c**) in 293T cells followed by Flag immunoprecipitation. (**d**) Detergent-sensitive association between DEPTOR and KDM4A. Expression of Flag-tagged mTOR or KDM4A in 293T cells followed by Flag immunoprecipitation using CHAPS or triton-based lysis buffer. (**e**) KDM4A-DEPTOR interaction is independent of mTOR. Depletion of mTOR by lentiviral infection of two different shRNAs in 293T cells transfected with Flag tagged KDM4A, followed by Flag immunoprecipitation. (**f**) KDM4A-DEPTOR interaction is independent of mSin1. Depletion of mSin1 by lentiviral infection in 293T cells transfected and immunoprecipitated as in (e). (**g**) Endogenous KDM4A and DEPTOR interact in HeLa cells. (**h**) Endogenous KDM4B and DEPTOR interact in 293E cells. (**i**) Recombinant GST-DEPTOR and His-KDM4A(1-562) or His-KDM4A(563-1064) do not interact *in vitro*.

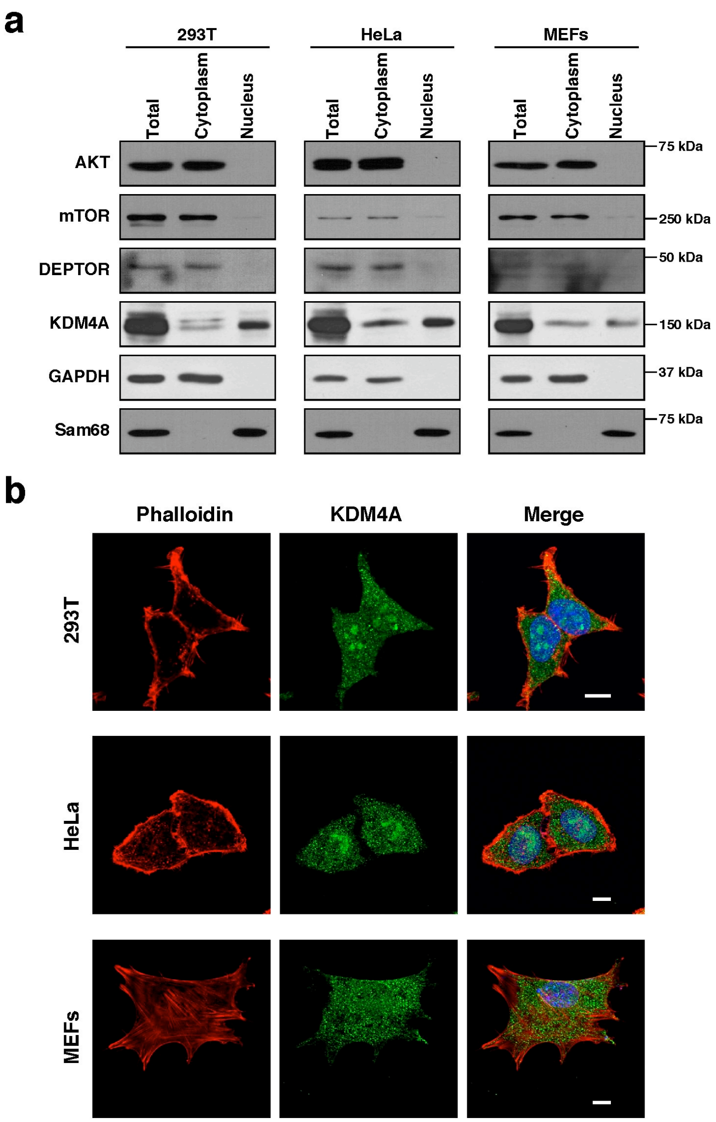

Supplementary Figure 4. KDM4A localized to both cytoplasm and nucleus. (**a**) Cell fractionation of 293T, HeLa, and MEFs followed by immunoblots against KDM4A, mTOR and DEPTOR. (**b**) Indirect immunofluorescence against KDM4A in 293T, HeLa and MEFs showing a nuclear and cytoplasmic distribution using confocal microscopy. Scale bars, 10 μm.

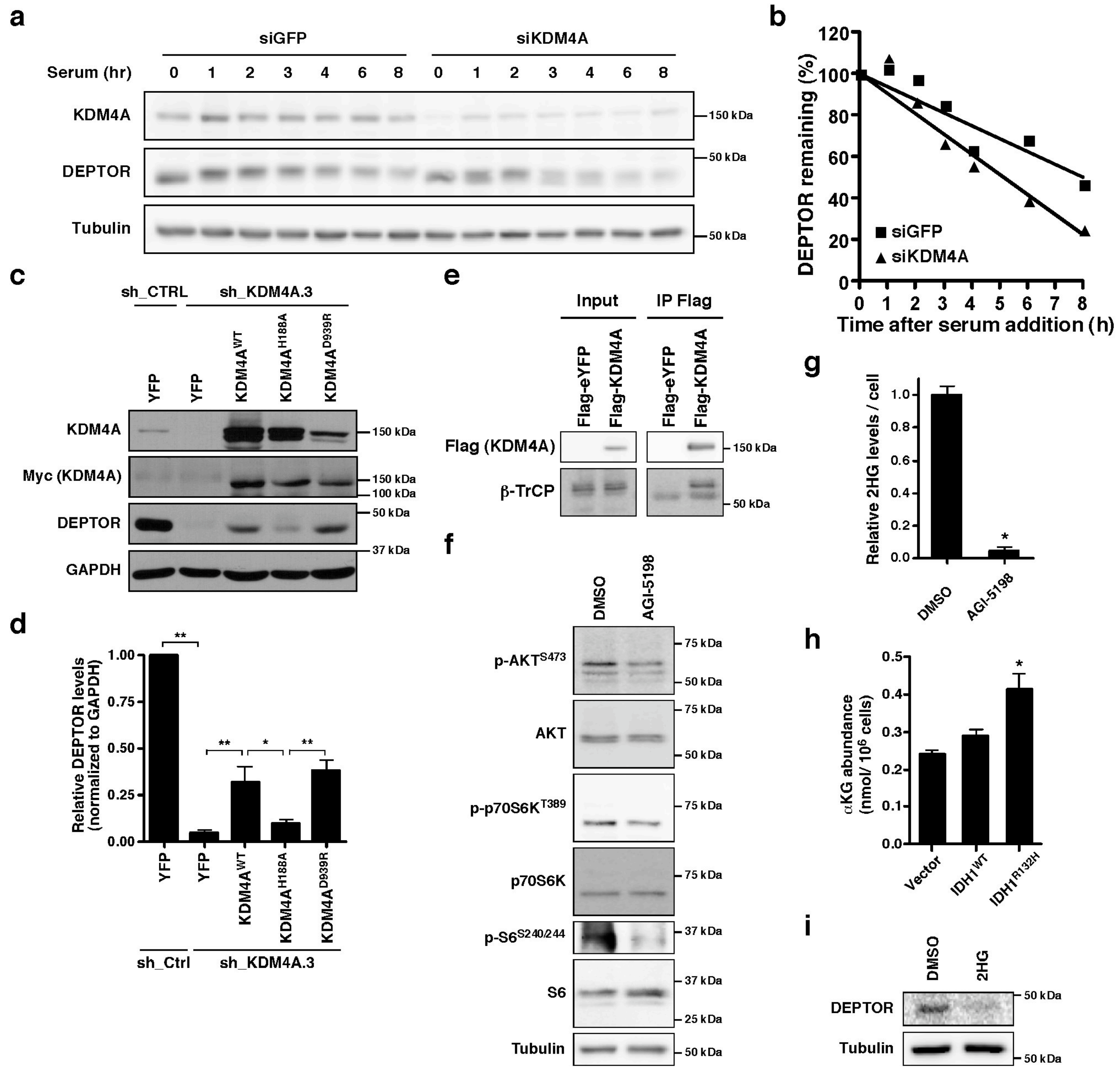

Supplementary Figure 5. Inhibition of endogenous IDH1 mutant with AGI-5198 decreases mTORC1/2 activity. (**a**) KDM4A depletion decreases DEPTOR protein stability. KDM4A was depleted in HeLa cells using siRNA. Cells were pre-treated with cycloheximide and chloramphenicol 1h prior to serum addition and whole cell lysates were blotted at different time points after serum stimulation. (**b**) Quantification of DEPTOR protein levels and normalized to tubulin, as shown in (a). (**c**) KDM4A catalytic activity is required to stabilize DEPTOR. Endogenous KDM4A was depleted in HeLa cells using sh_KDM4A.3. Cells were then transiently transfected with sh_RNA-resistant wild type KDM4A or mutants. (**d**) Quantification of three indepedent replicates of the rescue experiment performed as in (c). Statistical analysis was performed using two-sided *t*-test. * $P = 0.01$; ** $P < 0.01$. (**e**) Co-immunoprecipitation of endogenous β-TrCP with Flag-KDM4A in 293T transfected cells. (**f**) Inhibition of endogenous mutated $IDH1^{R132C}$ decreases mTOR activity. $IDH1^{R132C/WT}$ heterozygous HT1080 cells were treated for 3 days with 7.5 μM AGI-5198, a specific inhibitor of mutated IDH1. (**g**) AGI-5198-mediated inhibition of $IDH1^{R132C}$ decreases 2HG intracellular levels. HT1080 cells were treated with 3 μM AGI-5198 twice every day for 5 days, followed by determination of relative 2HG

levels. Asterisk denotes a statistical difference between AGI-5198 and DMSO-treated cells, two-sided *t*-test $P < 0.001$ (graph represents 3 independent experiments). Error bars represent standard deviation. (**h**) αKG abundance in NHA expressing empty vector control, $IDH1^{WT}$ or $IDH1^{R132H}$. Asterisk denotes a statistical difference between $IDH1^{R132H}$ and empty vector control cells, two-sided *t*-test $P = 0.04$ (graph represents 3 independent experiments). Error bars represent standard deviation. (**i**) Decreased DEPTOR levels in NHA treated with 1 mM 2HG during 8 hours.

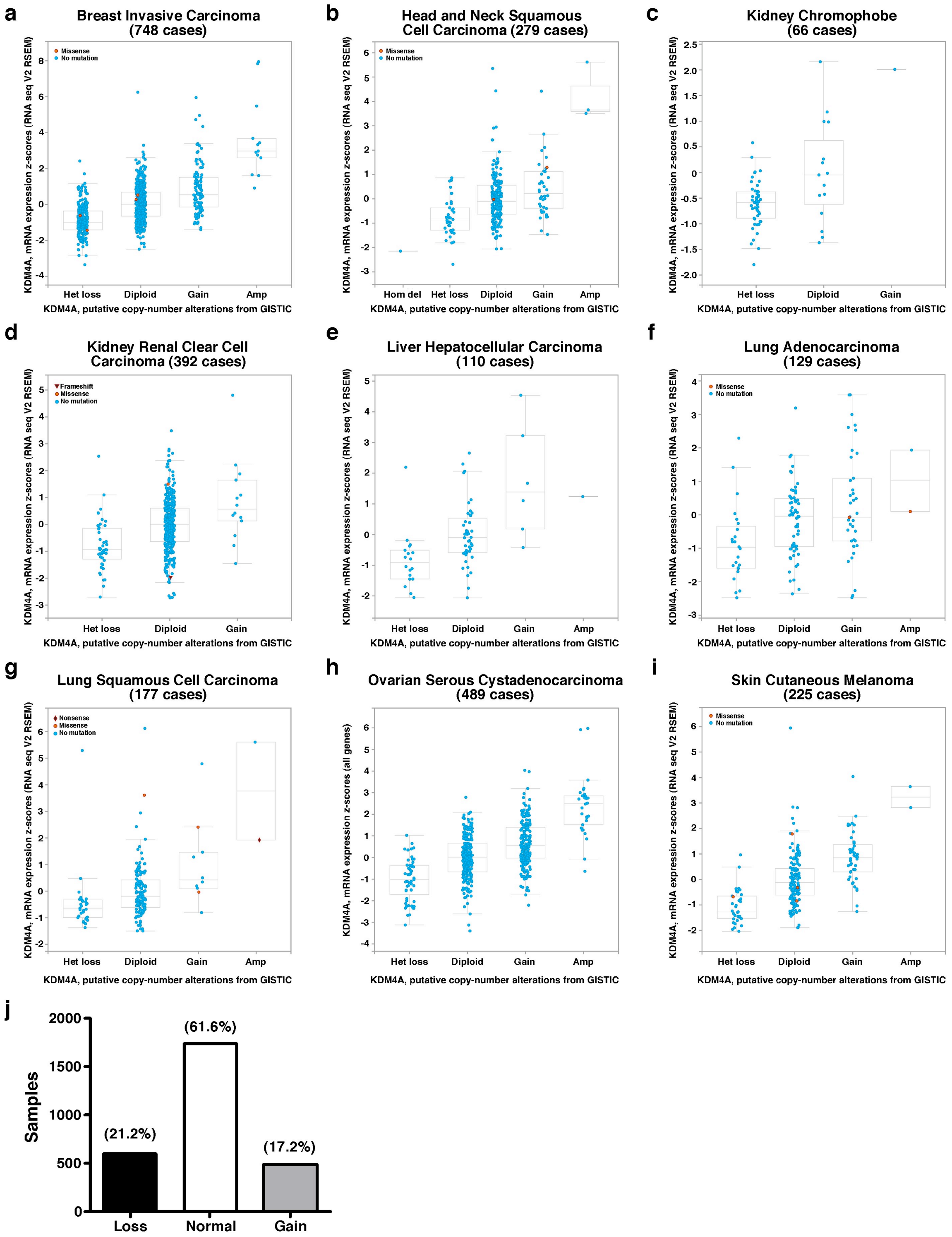

Supplementary Figure 6. Frequent genomic loss and decreased mRNA levels of *KDM4A* in various types of cancer. (**a-i**) Analysis of mRNA expression levels and gene copy number of *KDM4A* in breast invasive carcinoma (**a**), head and neck squamous cell carcinoma (**b**), kidney chromophobe (**c**), kidney renal clear cell carcinoma (**d**), liver hepatocellular carcinoma (**e**), lung adenocarcinoma (**f**), lung squamous cell carcinoma (**g**), ovarian serous cystadenocarcinoma (**h**) and skin cutaneous melanoma (**i**). (**j**) Loss (GISTIC annotation -2 and -1), normal (GISTIC annotation 0) and gain (GISTIC annotation +1 and +2) of copy number of *KDM4A* gene in 2812 cancer samples from TCGA and analysed with cBioPortal for Cancer Genomics [1,2].

Figure 1b

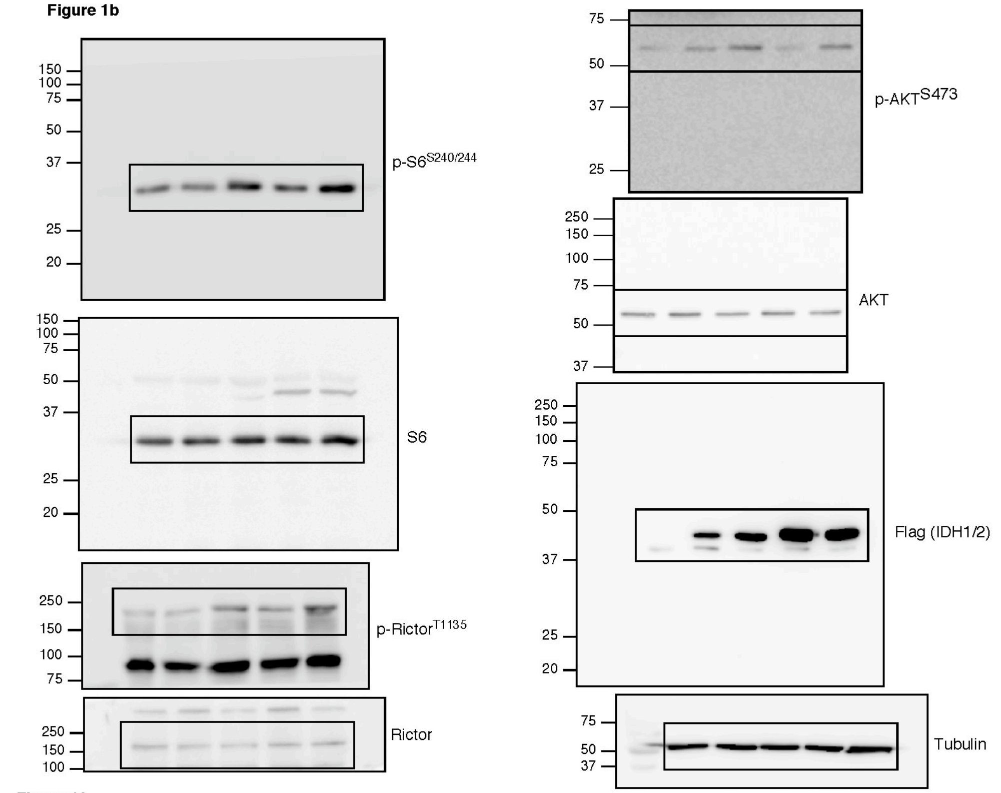

Figure 1f

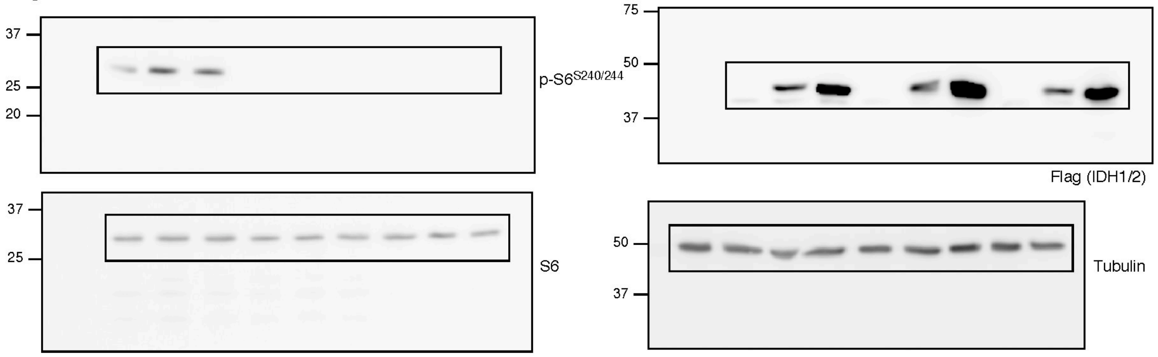

Figure 1g

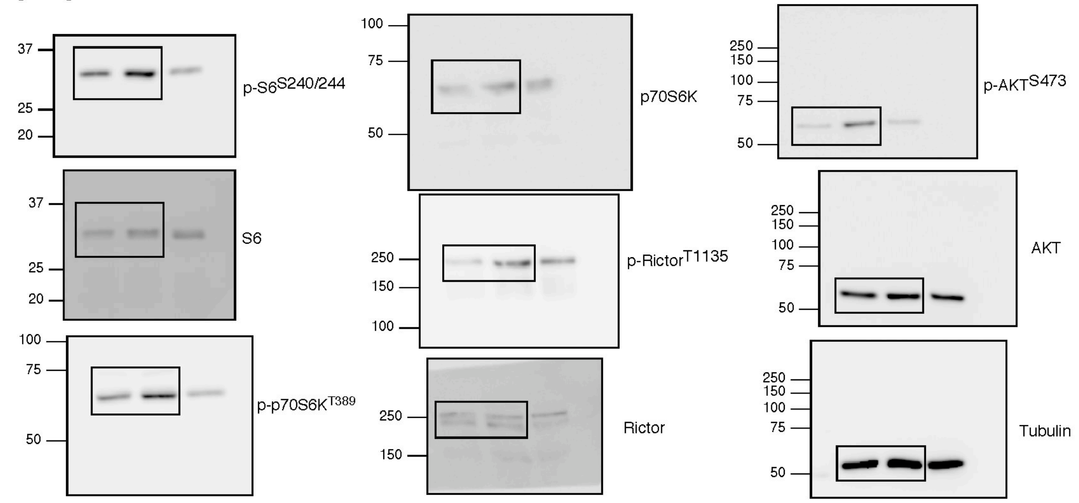

Supplementary Figure 7. (continued below)

Figure 2b

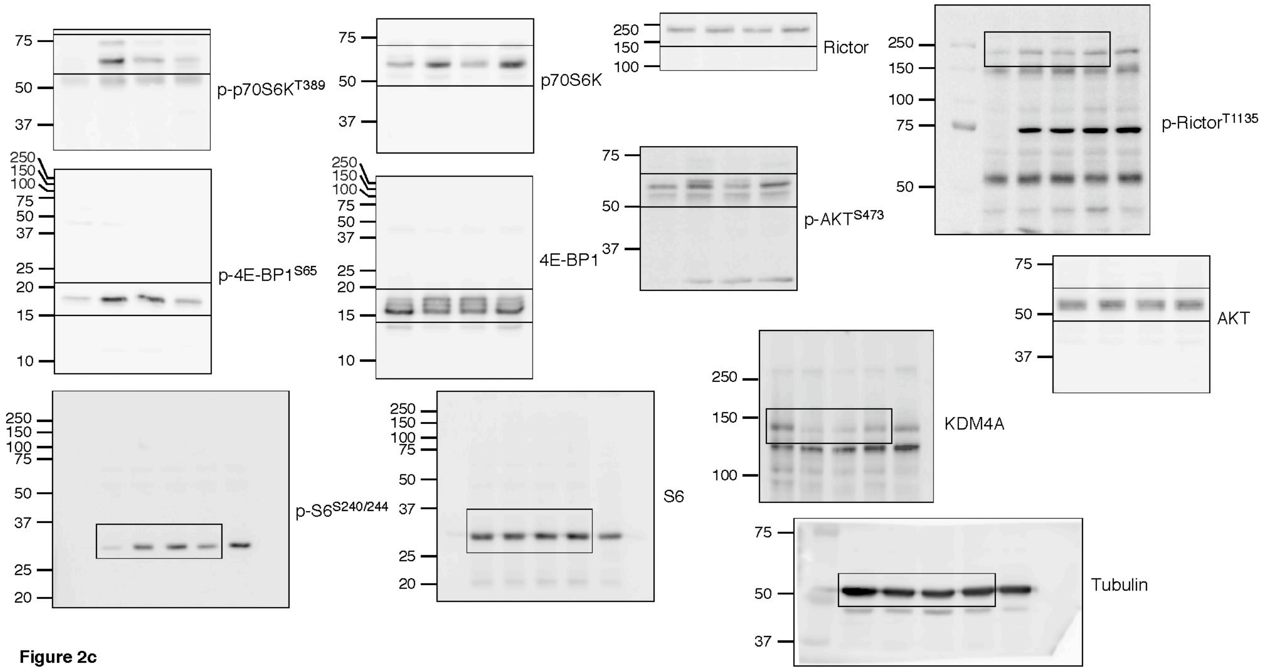

Figure 2c

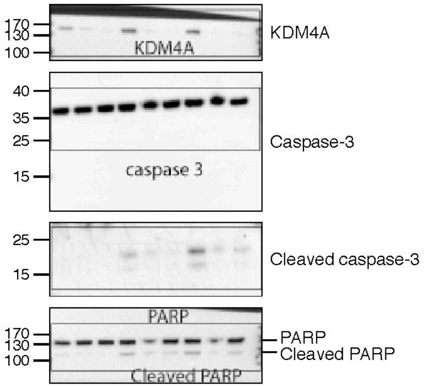

Figure 2f

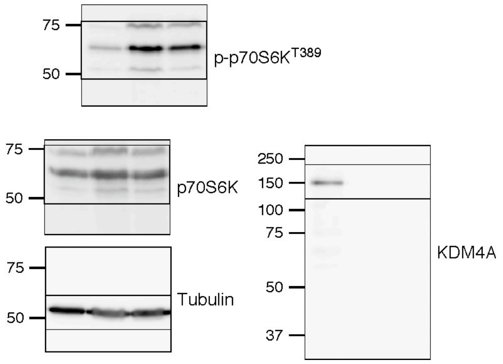

Supplementary Figure 7. (continued below)

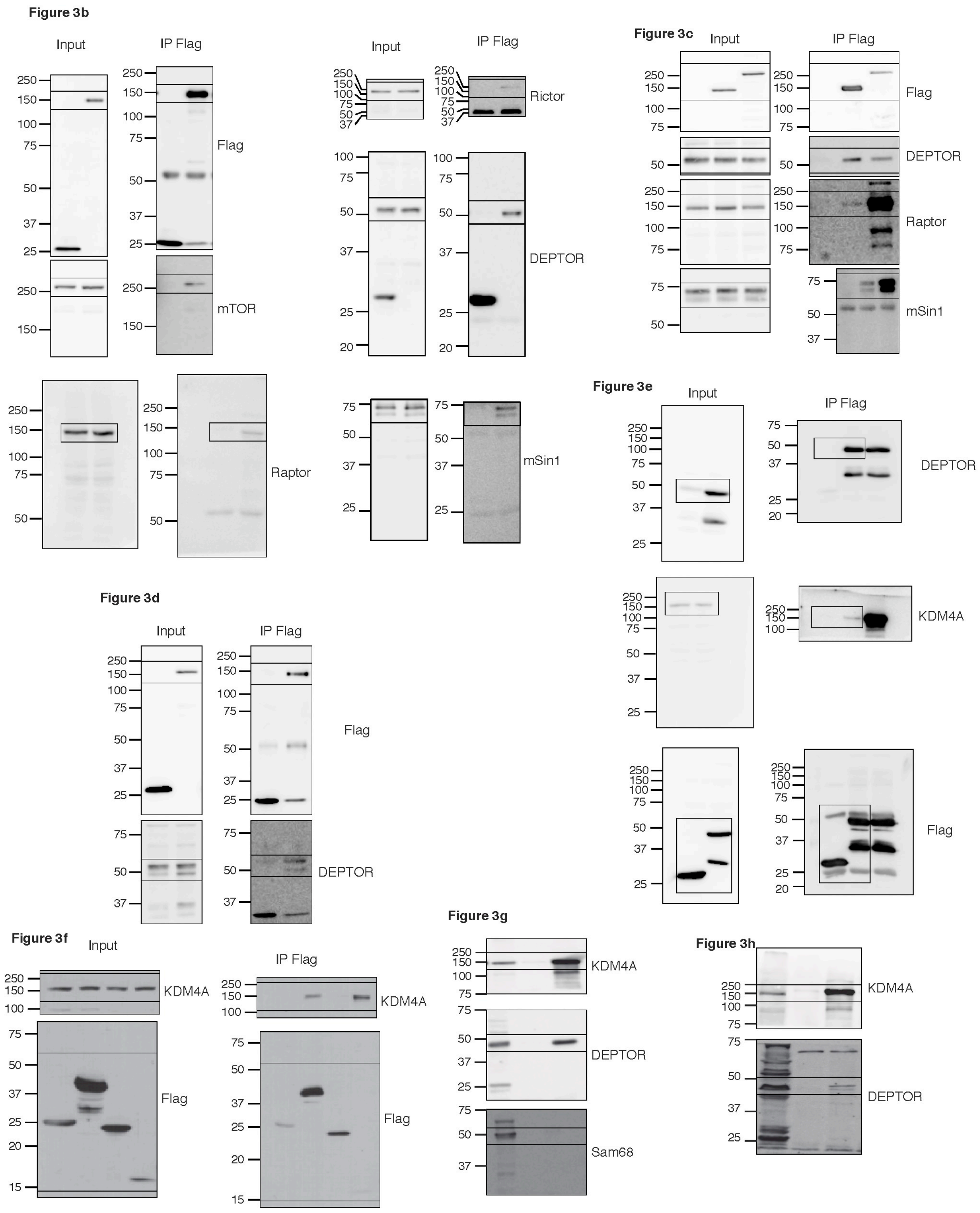

Supplementary Figure 7. (continued below)

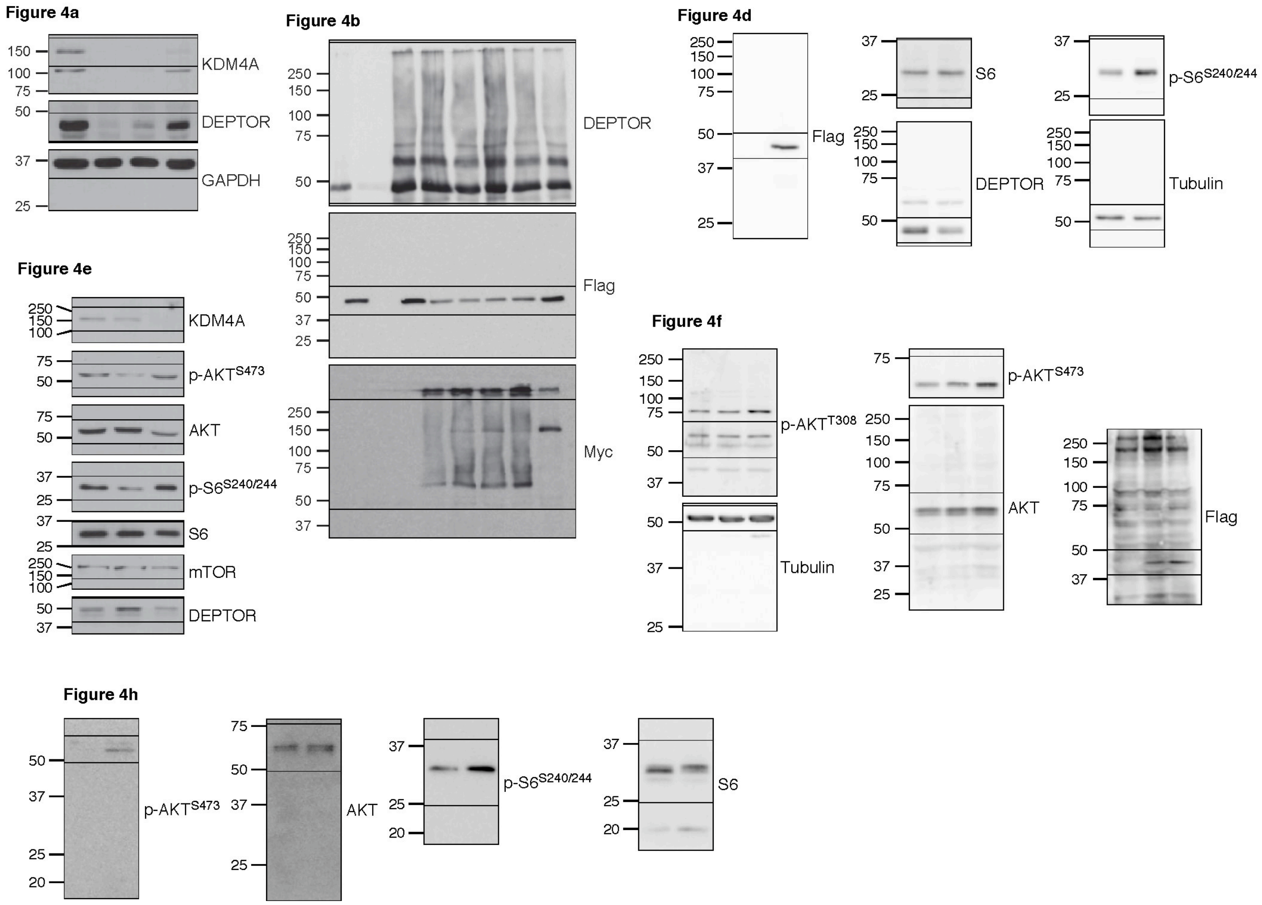

Supplementary Figure 7. (continued below)

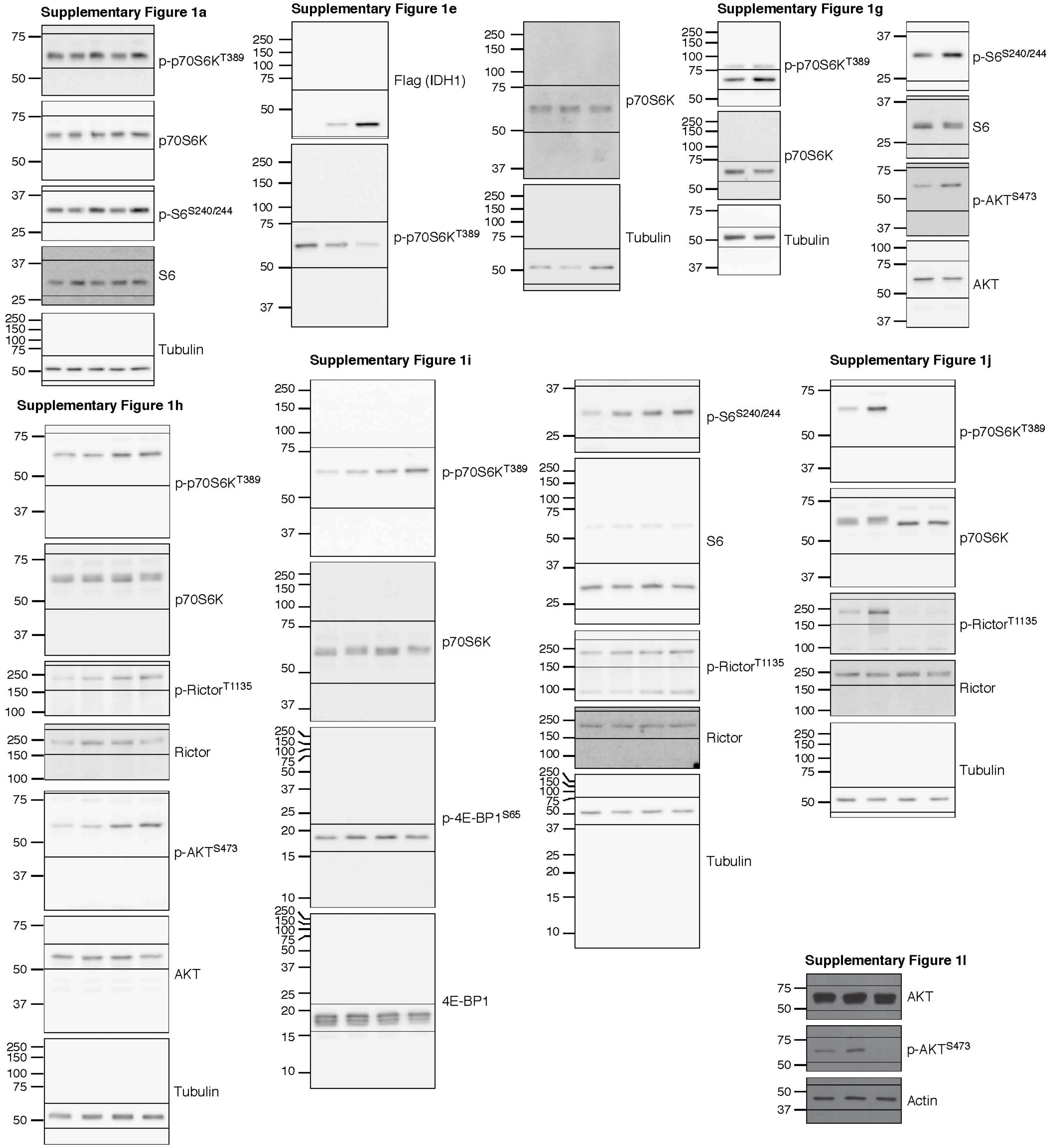

Supplementary Figure 7. (continued below)

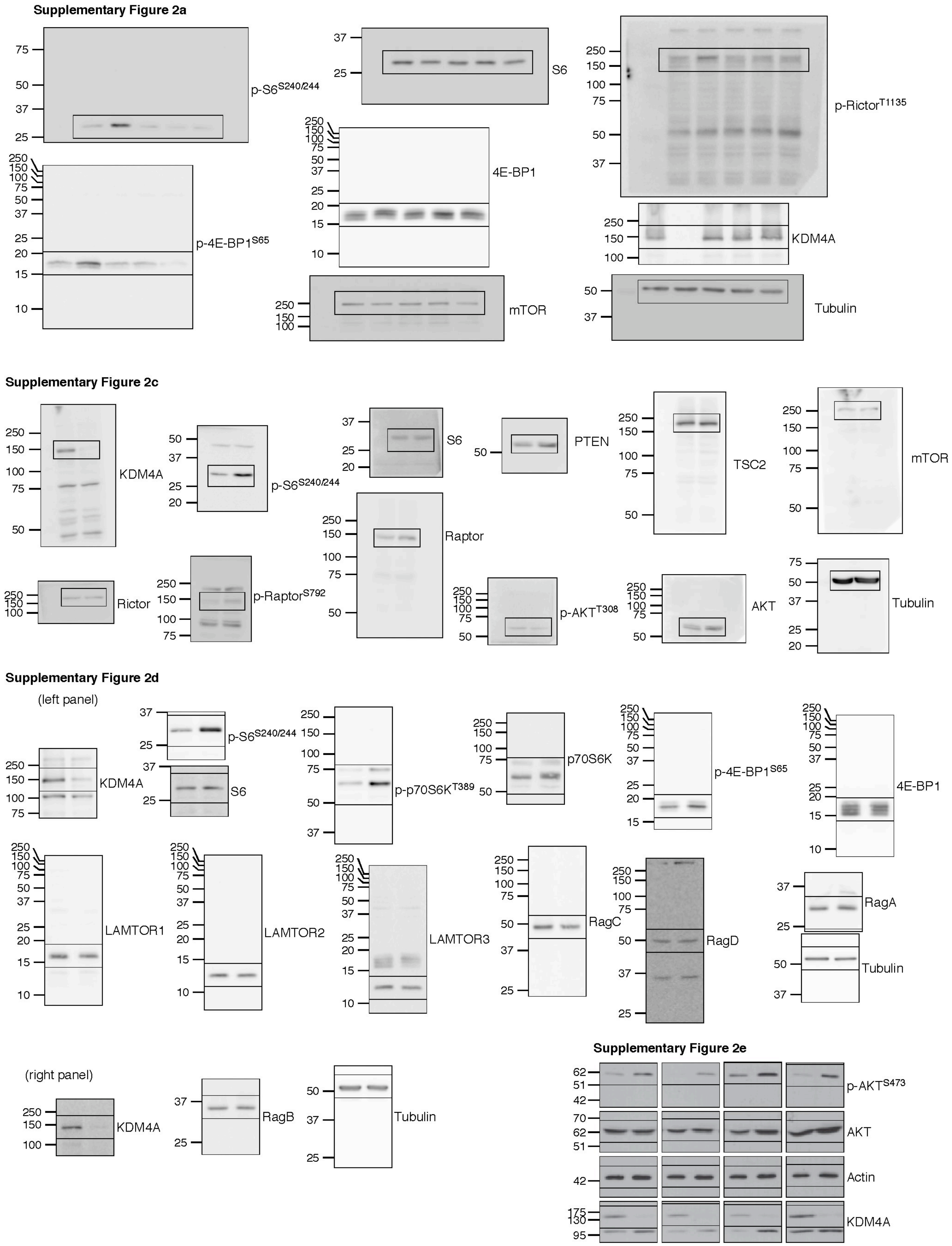

Supplementary Figure 7. (continued below)

Supplementary Figure 3a

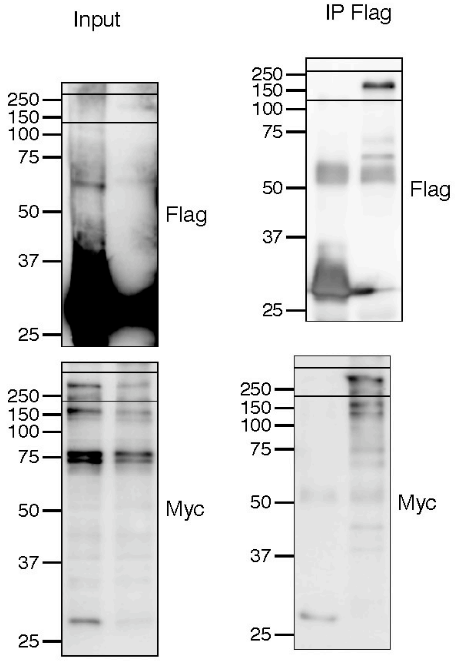

Supplementary Figure 3b

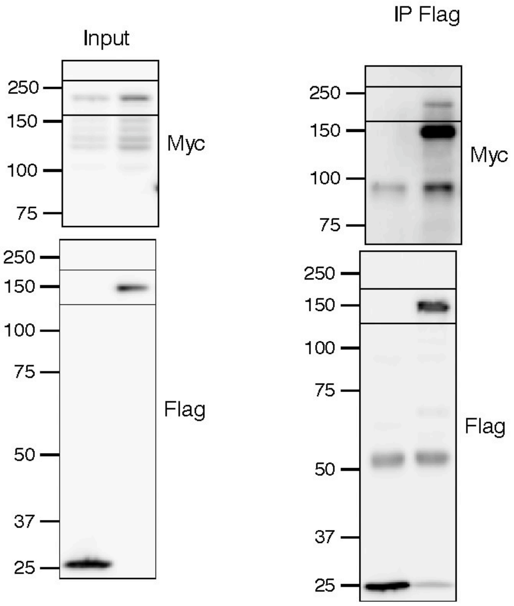

Supplementary Figure 3c

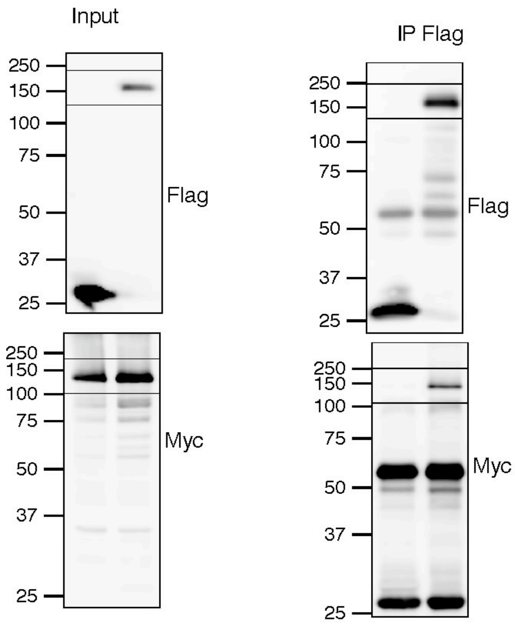

Supplementary Figure 7. (continued below)

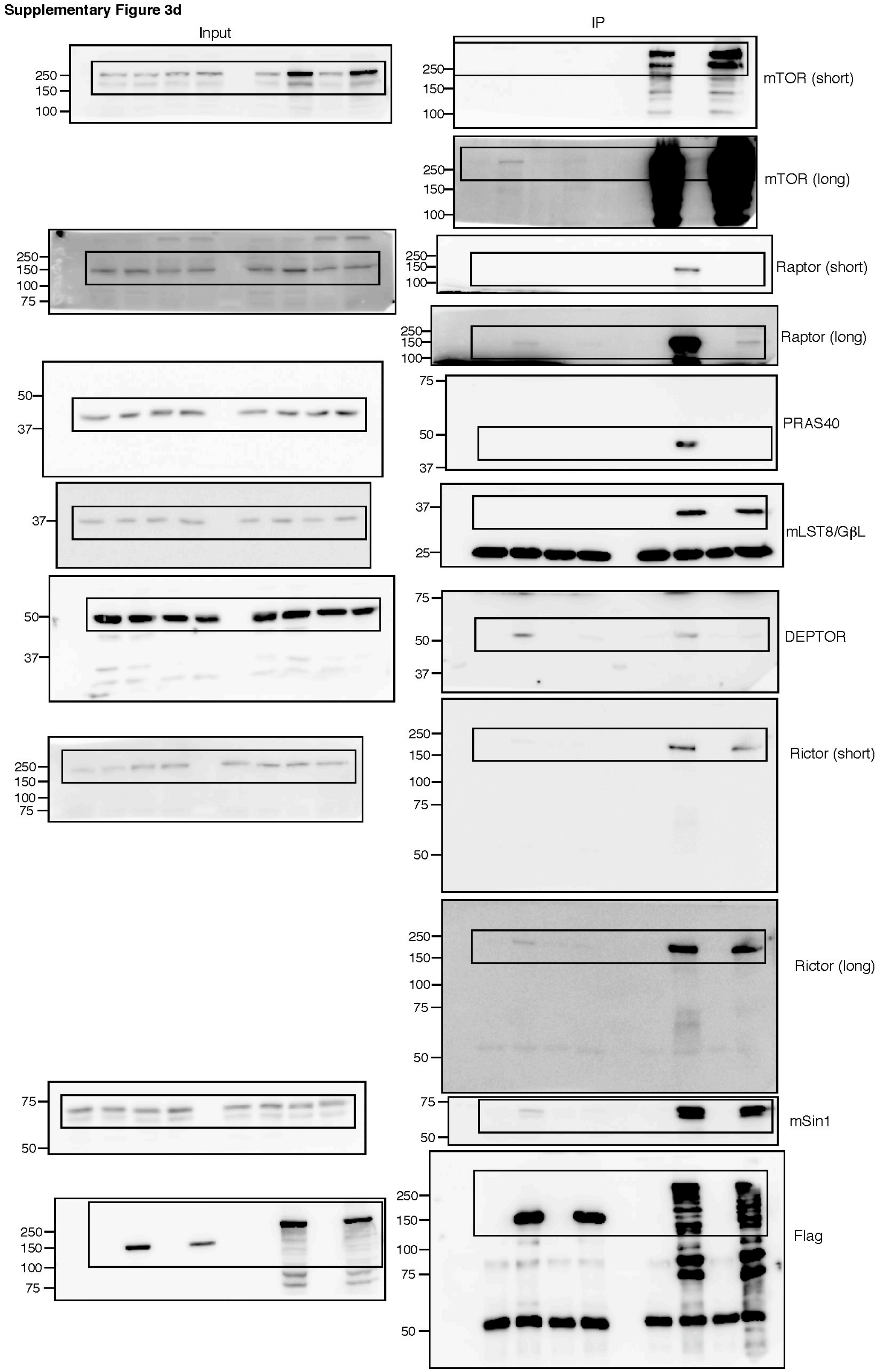

Supplementary Figure 7. (continued below)

Supplementary Figure 3e

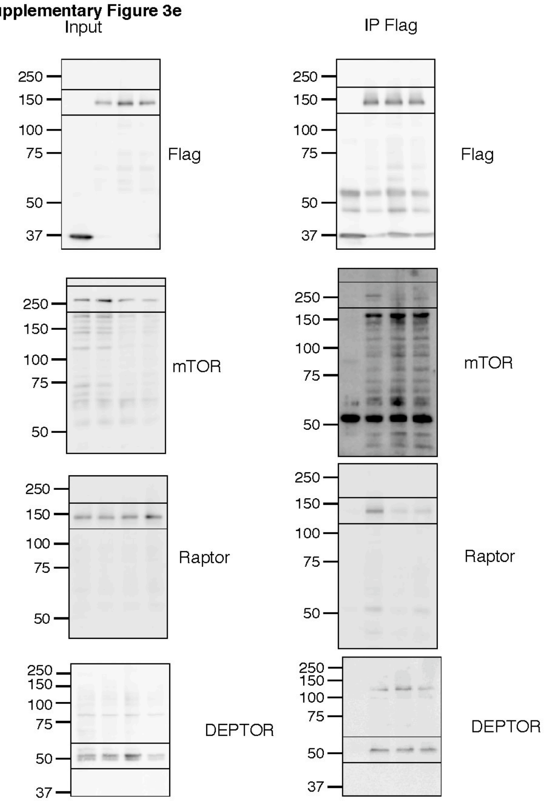

Supplementary Figure 3f

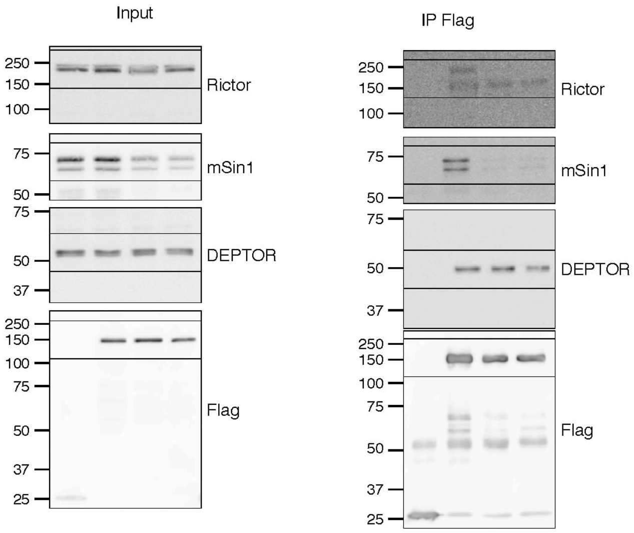

Supplementary Figure 3g

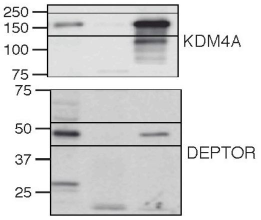

Supplementary Figure 3h

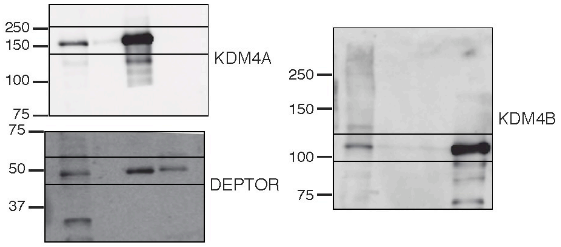

Supplementary Figure 3i

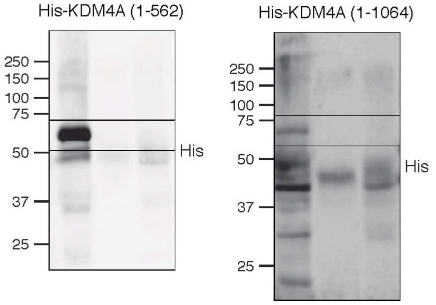

Supplementary Figure 7. (continued below)

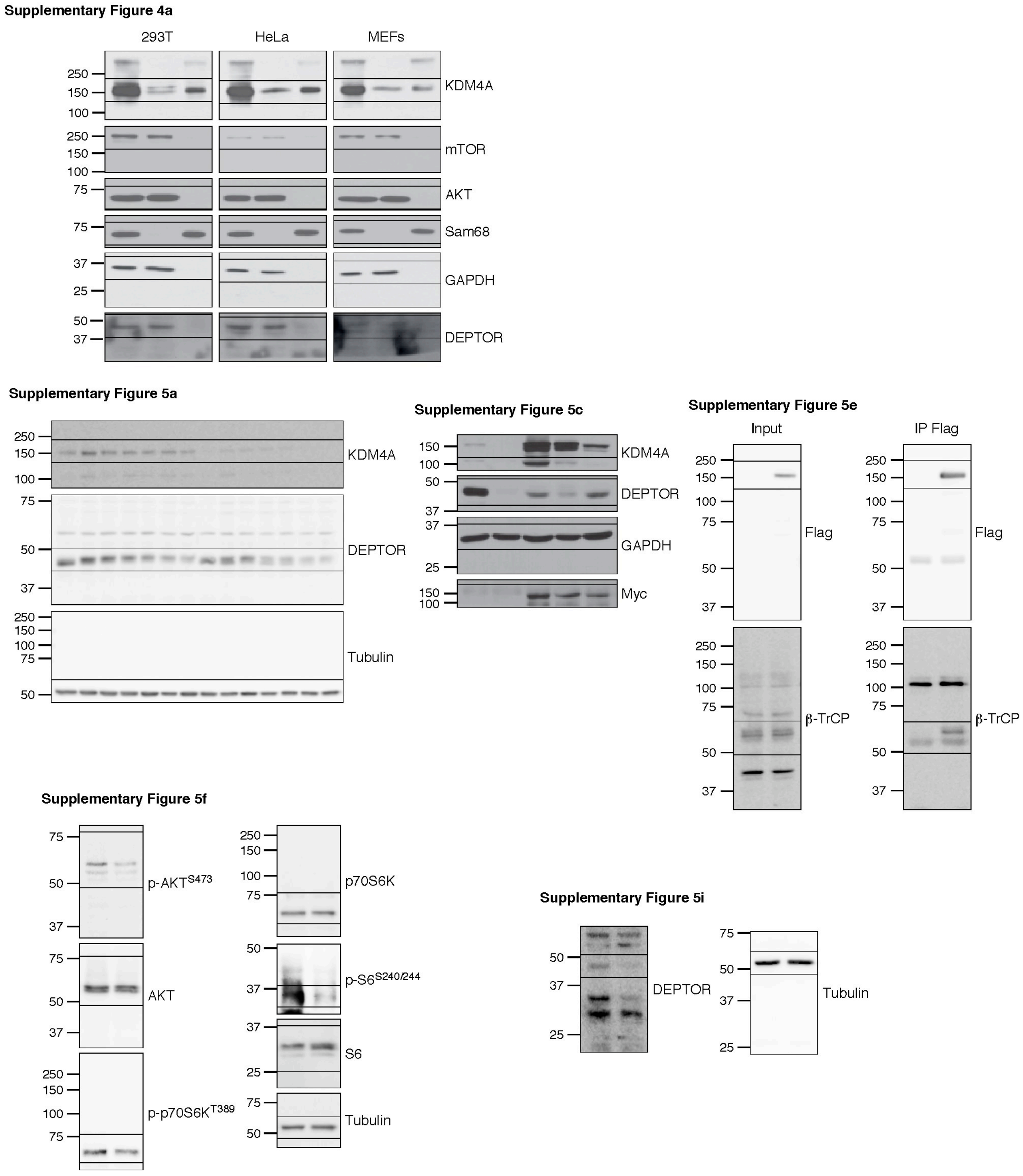

Supplementary Figure 7. Full scans of Western blots presented in the manuscript. Some images were re-scanned to cover larger area of the membrane, therefore causing slight differences in the contrast. Corresponding figures are indicated on top of the scans.

Supplementary Table 1. List of all siRNA Smart pools used in Fig. 2a.

#	Gene Symbol	Pool Catalog Number*	GENE ID	Gene Accession	GI Number
Ctrl	siGFP	n/a	n/a	n/a	n/a
1	KDM2A/JHDM1A/FBXL11	M-012458-00	22992	NM_012308	16306579
2	KDM2B/JHDM1B/FBXL10	M-014930-01	84678	NM_001005366	54112379
3	JHDM1C/FBXL19	M-031874-01	54620	NM_001099784	157168348
4	KDM3A/JMJD1A	M-017301-01	55818	NM_018433	156602657
5	KDM3B/JMJD1B	M-020378-01	51780	NM_016604	54873608
6	JMJD1C	M-012686-01	221037	NM_004241	68342035
7	KDM4A/JMJD2A	M-004292-01	9682	NM_014663	98986458
8	KDM4B/JMJD2B	M-004290-01	23030	NM_015015	45504379
9	KDM4C/JMJD2C	M-004293-01	23081	NM_015061	109255246
10	KDM4D/JMJD2D	M-020709-00	55693	NM_018039	39653316
11	KDM4E/Loc390245/JMJD2E	M-029951-01	390245	XM_372429	113422553
12	KDM5A/JARID1A	M-003297-03	5927	NM_001042603	110618243
13	KDM5B/JARID1B	M-009899-01	10765	NM_006618	57242795
14	KDM5C/JARID1C	M-010097-01	8242	NM_004187	109255242
15	KDM5D/JARID1D	M-010820-01	8284	NM_004653	56243542
16	KDM6A/UTX	M-014140-01	7403	NM_021140	10863942
17	KDM6B/JMJD3	M-023013-01	23135	NM_001080424	122937250
18	UTY	M-017344-00	7404	NM_007125	33188430
19	KDM7A/JHDM1D/KIAA1718	M-025357-01	80853	NM_030647	90093354
20	KDM8/JMJD5	M-003983-02	79831	NM_024773	13376122
21	JMJD4	M-014238-00	65094	NM_023007	12711669
22	JMJD6/PTDSR	M-010363-03	23210	NM_015167	125988388
23	JMJD8/loc3391123	M-022873-01	339123	NM_001005920	56090145
24	JARID2	M-009244-01	3720	NM_004973	11863151
25	PHF2	M-012912-01	5253	NM_005392	117190341
26	PHF8	M-004291-00	23133	NM_015107	32698699
27	HR	M-011872-01	55806	NM_018411	70906481
28	NO66/c14orf169	M-014397-01	79697	NM_024644	106879205
29	MINA/NO52	M-016031-01	84864	NM_153182	110227620
30	HIF1AN	M-004073-02	55662	NM_017902	148596935
31	HSPBAP1	M-004287-01	79663	NM_024610	142383186
32	FTO	M-004159-01	79068	NM_001080432	122937262
33	LSD1/AOF1	M-008121-01	221656	NM_153042	116256450
34	LSD2/AOF2	M-009223-01	23028	NM_015013	58761545
35	TET2	SASI_Hs02_00328772;<br>SASI_Hs02_00328773;<br>SASI_Hs02_00328774;<br>SASI_Hs02_00328775.	54790	NM_001127208	187761317
36	TET3	SASI_Hs02_00364816;<br>SASI_Hs02_00364817;<br>SASI_Hs02_00364818;<br>SASI_Hs02_00364819.	200424	NM_144993	149944516

* All siRNAs were obtained from ThermoFisher/Dharmacon, except for TET2 and TET3 which were from Sigma-Aldrich.

Supplementary Table 2. List of primers used for RT-qPCR using the Universal Probe Library assay.

Gene name	Forward primer	Reverse primer	UPL probe
**KDM4A**	gccgctagaagtttcagtgag	gcgtcccttggacttcttatt	53
**PTEN**	ggggaagtaaggaccagagac	tccagatgattctttaacaggtagc	48
**REDD1**	ctggacagcagcaacagtg	acaccccatccaggtaagc	69
**TSC1**	caaccagagccaggaattaca	cagctccgcaatcatgttc	65
**TSC2**	actgctggagttcctgtcca	gcggcaaagttcctgtaga	64
**DEPTOR**	ctttgccaccggcttatg	tccacaaatgggtgcttgt	42
**Rheb**	tgggttacagctgattgaagc	tcatcactggggaggaactc	81
**mTOR**	tttagcggtcatgtcaatgg	catcaggttggatgggtgt	14
**PDK1**	gttcatgtcacgctgggtaa	tgaaaagtctgtcaattttcctca	10
**KDM4B**	ggcctcaagtgacgagga	cttccactgcagagacagca	67
**KDM4C**	aggcgccaagtgatgaag	gagaggtttcgcccaagact	69
**KDM4D**	gccgatctgcagttagtgg	gggcttcttagagctgtgga	66
**HPRT**	tgatagatccattcctatgactgtaga	caagacattctttccagttaaagttg	22
**ACTB**	attggcaatgagcggttc	tgaaggtagtttcgtggatgc	11
**GAPDH**	agccacatcgctcagacac	gcccaatacgaccaaatcc	60

Supplementary References

1. Cerami, E. *et al.* The cBio cancer genomics portal: an open platform for exploring multidimensional cancer genomics data. *Cancer Discov* **2**, 401-404 (2012).
2. Gao, J. *et al.* Integrative analysis of complex cancer genomics and clinical profiles using the cBioPortal. *Sci Signal* **6**, pl1 (2013).
